# Hepatocyte-Specific MET Deletion Exacerbates Acetaminophen-Induced Hepatotoxicity in Mice

**DOI:** 10.1016/j.ajpath.2025.09.010

**Published:** 2025-09-30

**Authors:** Siddhi Jain, Ranjan Mukherjee, Gillian Williams, Jia-Jun Liu, Lanuza A.P. Faccioli, Zhiping Hu, Rodrigo M. Florentino, George K. Michalopoulos, Alejandro Soto-Gutierrez, Silvia Liu, Joseph Locker, Bharat Bhushan

**Affiliations:** ∗ Department of Pathology, University of Pittsburgh School of Medicine, Pittsburgh, Pennsylvania; † Pittsburgh Liver Research Center, University of Pittsburgh, Pittsburgh, Pennsylvania; ‡ Organ Pathobiology and Therapeutics Institute, University of Pittsburgh School of Medicine, Pittsburgh, Pennsylvania; § Department of Pharmacology and Chemical Biology, University of Pittsburgh School of Medicine, Pittsburgh, Pennsylvania; ¶ Center for Transcriptional Medicine, University of Pittsburgh, Pittsburgh, Pennsylvania

## Abstract

Despite the well-known role of MET in liver regeneration after partial hepatectomy, its role in the clinically relevant acetaminophen (APAP)-induced liver injury (AILI) model remains unexplored. AILI markedly differs from partial hepatectomy because it is associated with massive liver necrosis. This study aims to delineate the role of MET specifically in AILI. Hepatocyte-specific MET knockout (MET KO) mice were administered a toxic dose of APAP and assessed for liver injury/regeneration parameters. MET deletion strikingly exacerbated the initial hepatotoxicity and consequentially impaired the compensatory proliferative response, culminating in significant mortality. Mechanistically, MET deletion enhanced c-Jun N-terminal kinase (JNK) activation and its mitochondrial translocation, resulting in excessive mitochondrial oxidative damage, releasing apoptosis-inducing factor into cytosol. Excess JNK activation was attributed to reduced inhibitory activity of AKT on JNK in the absence of MET signaling. Pharmacologic activation of AKT reduced JNK activation and hepatotoxicity in MET KO mice. RNA-sequencing/immunoblotting not only showed repression of proliferative/survival signaling but also activation of cell death/senescence pathways along with an impaired unfolded protein response in MET KO mice. Analysis of published single-nucleus RNA-sequencing data showed that proliferation in livers from patients with APAP-induced acute liver failure was associated with strong activation of hepatocyte growth factor/MET signaling in hepatocytes, with spatial transcriptomics showing striking induction of hepatocyte growth factor surrounding the necrotic zones. Interestingly, 35% of the genes altered in human acute liver failure were regulated by MET in the mouse AILI model. The current study shows that MET is crucial for restraining hepatotoxicity after APAP overdose via inhibition of the mitochondrial cell death signaling pathway.

## Graphical Abstract

**Figure:**
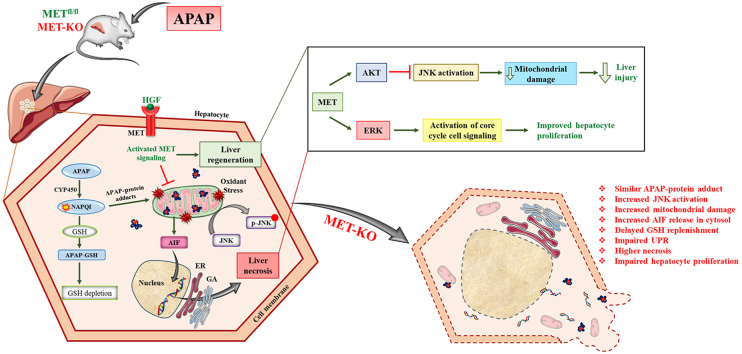

Acetaminophen (APAP) overdose is the leading cause of acute liver failure (ALF) in the United States, responsible for >46% of all ALF cases and approximately 80,000 cases presenting to hospitals annually.^1^^,^^2^ Current treatment options are limited, with *N*-acetylcysteine as the only available pharmacologic treatment. However, *N*-acetylcysteine is effective only within a narrow time window, and liver transplantation is often the final option.^2^^,^^3^ APAP-induced liver injury (AILI) involves formation of a reactive metabolite, *N*-acetyl-*p*-benzoquinone imine (NAPQI), which binds to cellular proteins, predominantly in mitochondria, inducing oxidative stress. Excessive mitochondrial oxidative damage causes release of DNA damaging factors, eventually leading to centrilobular necrosis.^4^ APAP-induced liver necrosis is followed by compensatory liver regeneration, which determines the final recovery.^3^^,^^5^ Unlike surgical resection of healthy liver [ie, partial hepatectomy (PHx)], AILI causes extensive liver damage and inflammation, which markedly alter the dynamics of regenerative processes.^6^

Hepatocyte growth factor (HGF), a pivotal mitogen for hepatocytes, is important for liver regeneration through activation of its receptor MET. Together with epidermal growth factor receptor, MET serves as one of the two most crucial regulators of the regenerative process after PHx.^7^ Although simultaneous elimination of both these signals results in the complete abolition of liver regeneration after PHx,^8^ deletion of MET alone results only in delayed regeneration and recovery.^8^^,^^9^ In addition to the regenerative function of MET signaling, prior research has indicated its role in apoptosis signaling and maintaining redox balance.^10^ Although the role of MET in APAP-induced programmed liver necrosis and ensuing compensatory liver regeneration remains poorly understood, it is critical to explore, given the high clinical relevance of AILI.

In an earlier study, a dose-dependent activation of MET was observed in response to APAP overdose in mice closely preceding liver injury development, indicating a potential role of MET in APAP-induced hepatotoxicity beyond its regenerative role.^11^ It is crucial to investigate the causal role of MET specifically in AILI, which cannot be presumed identical to its role in regeneration of healthy liver after resection. The necessity to carefully examine the role of MET in AILI is also underscored by a previous study, which showed a paradoxical injury-promoting facet of epidermal growth factor receptor in AILI, even though epidermal growth factor receptor closely resembles MET in the context of liver regeneration function.^12^ The current study shows for the first time that in a murine AILI model, hepatocyte-specific deletion of MET significantly aggravates APAP hepatotoxicity and hinders subsequent compensatory proliferative response. Furthermore, it uncovers the mechanisms through which MET inhibits c-Jun N-terminal kinase (JNK) activation and its mitochondrial translocation to limit mitochondrial damage and subsequent hepatic necrosis after APAP overdose. These findings emphasize the crucial protective role of MET signaling beyond promoting hepatocyte regeneration in restricting hepatotoxicity after APAP overdose.

## Materials and Methods

### Animals, Treatments, and Tissue Collection

Mice with a MET^flox/flox^ genotype were used, similarly to earlier studies^8^^,^^13^; characterization of these mice has been described previously.^14^ To obtain hepatocyte-specific MET knockout (MET KO) and wild-type (WT) control mice, 7- to 8-week–old male MET^flox/flox^ mice were administered intraperitoneally the vectors AAV8.TBG.PI.Cre.rBG (viral prep #107787-AAV8; Addgene, Watertown, MA) and AAV8.TBG.PI.eGFP. WPRE.bGH8 (viral prep #105535-AAV8; Addgene), respectively, at a dose of 2.5 × 10^11^ viral particles per mouse.^15^, ^16^, ^17^ One week after administering the AAV8 vector injections, mice were used for subsequent experimental procedures.

The effectiveness of gene knock-down was assessed by direct examination of RNA-sequencing data from these mice (Supplemental Figure S1). Mice were subjected to 16 hours of overnight fasting before receiving an i.p. injection of 300 mg/kg APAP (Sigma Aldrich, St. Louis, MO) dissolved in warm 0.9% saline, and food was returned to the mice 2 hours after APAP administration. Blood and liver samples from WT and MET KO mice (*n* = 3 to 6) were collected at 0, 1, 3, 6, and 24 hours after APAP administration following cervical dislocation under isoflurane anesthesia.

To investigate if AKT repression is involved in aggravated JNK activation and liver injury in MET KO mice, an AKT activator (SC79: 10 mg/kg, i.p.) (#HY-18749; MedChemExpress, Monmouth Junction, NJ) was used in the MET KO mice. SC79 or its vehicle (4% dimethyl sulfoxide) was given 2 hours’ post-APAP overdose, and liver tissue was collected at 6 and 24 hours. A dose of 600 mg/kg APAP was used for all AKT activator studies to induce a severe injury phenotype in MET KO mice as the vehicle used contained dimethyl sulfoxide, a known suppressor of APAP metabolic activation and AILI.^18^

All experimental procedures complied with the standards of the University of Pittsburgh Association for Assessment and Accreditation of Laboratory Animal Care accredited facilities. The animals were housed under stringent conditions, including a 12 hours’ light/dark cycle, and all protocols were approved by the Institutional Animal Care and Use Committee.

### Primary Human Hepatocyte Experiments

Freshly isolated normal primary human hepatocytes were obtained from the Human Liver Tissue & Hepatocytes Research Resource (Pittsburgh, PA), which was funded by the National Institute of Diabetes and Digestive and Kidney Diseases project 1R24DK139775. Primary human hepatocytes were suspended in hepatocyte maintenance medium (#CC3197; Lonza, Rockland, ME) supplemented with dexamethasone, insulin, gentamicin sulfate-amphotericin, and 10% fetal bovine serum, and seeded onto collagen-coated six-well plates. Next day, the hepatocytes were washed with sterile phosphate-buffered saline and treated for 24 hours with 10 mM APAP alone or in combination with 0.1 or 1 μM capmatinib dihydrochloride hydrate (a MET inhibitor) (#HY-13404C; MedChemExpress) in fetal bovine serum–free media in the presence of HGF (50 ng/mL). After treatment, cells were stained with propidium iodide to assess cell death. All experiments were replicated with at least three separate cell isolations.

### Histologic Analysis and Serum Alanine Aminotransferase/Aspartate Aminotransferase Measurements

The collected blood samples underwent serum separation to assess alanine aminotransferase and aspartate aminotransferase levels. Paraffin-embedded liver sections, 4 μm thick, were deparaffinized and treated with xylene, followed by hydration through a series of graded alcohol solutions and distilled water. The slides were then stained with hematoxylin for 1 minute, washed under tap water, and stained with eosin for 15 seconds. After washing, the sections were dehydrated, mounted with Cytoseal (#8310-4; Epredia, Kalamazoo, MI), and scored for the percentage of necrotic area.

### Electron Microscopy

The liver tissues were initially fixed in ice-cold Karnovsky fixative (2.5% glutaraldehyde in phosphate-buffered saline), then in 1% osmium tetroxide with 1% potassium ferricyanide, followed by phosphate-buffered saline rinsing. The samples were dehydrated using a graded ethanol and propylene oxide series and embedded in Poly/Bed 812 resin (Glauert formulations; Polysciences, Inc., Warrington, PA). Ultrathin 65 nm sections were stained with uranyl acetate and Reynold’s lead citrate, then imaged using a JEOL 1400 Plus transmission electron microscope equipped with an AMT 2k digital camera (Advanced Microscopy Techniques, Danvers, MA). Individual mitochondria were identified, counted, and traced to quantify their area using ImageJ software version 1.54p (NIH, Bethesda, MD; *https://imagej.net/ij*).^19^

### Estimation of Total Glutathione Level

The hepatic glutathione (GSH) levels were measured by using a GSH assay kit (Sigma Aldrich). The assay procedure was performed in accordance with the manufacturer’s instructions.

### Mitochondrial Isolation

The mitochondria were isolated from freshly harvested liver tissues of mice using the Mitochondria Isolation Kit for Tissue (#89801; Thermo Scientific, Waltham, MA) following the detailed protocol provided by the manufacturer. The isolated mitochondria were then digested using 2% CHAPS hydrate (#C5070; Sigma Aldrich) in Tris-buffered saline (25 mM Tris, 0.15 M NaCl; pH 7.2). The cytosolic lysates obtained during the mitochondrial isolation process, along with the mitochondrial fractions, were subsequently used for protein estimation and immunoblotting analysis.

### Protein Isolation and Immunoblotting

The total liver lysate used for the immunoblotting assay was prepared from frozen liver tissues using Tissue Protein Extraction Reagent (#78510; Thermo Scientific) with a protease and phosphatase inhibitor cocktail (#1861284; Thermo Scientific). The protein estimation was performed by using the bicinchoninic acid method according to the manufacturer’s protocol. An equal amount of protein was then separated on NuPAGE 4–12% Bis-Tris gels with 1X MOPS running buffer (Thermo Fisher Scientific, Waltham, MA). The separated proteins were transferred onto a Immobilon-P PVDF transfer membrane (#IPVH00010; Millipore, Burlington, MA), followed by blocking with Protein-Free blocking buffer for 30 minutes, and incubated overnight at 4°C with primary antibodies against phospho-JNK (T183/Y185; #4668), JNK (#9252), phospho-AKT (T308; #2965), AKT (#9272), GCLm (#33381), AIF (#4642), phospho-ERK1/2 (T202/Y204; #9101), ERK1/2 (#4696), phospho-RB (S807/811; #8516), Cyclin-D1 (#55506), PCNA (#2586), IRE1α (#3294), BiP (#3177), VDAC (#4661), GAPDH (#5174), and β-actin (#4970) from Cell Signaling Technology (Danvers, MA); p21 (#sc-6246) from Santa Cruz Biotechnology (Dallas, TX); TGF-β1 (#RDI-TGFβ1 abr) from R&D Systems (Minneapolis, MN); CYP2E1 (HPA009128) from Atlas Antibodies (Stockholm, Sweden); Phospho-IRE1 (S724; #AP1442) and GCLc (#A25132) from ABclonal Technologies (Woburn, MA). This was followed by incubation with a secondary antibody for 2 hours, and chemiluminescence was visualized by using the ChemiDoc Touch Imaging System (Bio-Rad, Hercules, CA). The visualized bands were quantified using densitometric analysis with ImageJ software version 1.54p (NIH, Bethesda, MD; *https://imagej.net/ij*) for relative quantification.

### Immunohistochemistry and Terminal Deoxynucleotidyl Transferase-Mediated dUTP Nick-End Labeling Assay

Paraffin-embedded liver sections (4 μm) were used for immunohistochemical evaluation of proliferating cell nuclear antigen (cell proliferation) following established protocols.^20^ The proliferating cell nuclear antigen antibody (1:3000, #2586; Cell Signaling Technology) was used for immunohistochemistry. DNA fragmentation was assessed by using a terminal deoxynucleotidyl transferase-mediated dUTP nick-end labeling (TUNEL) assay kit (#S7100; Sigma Aldrich) according to the instructions provided by the manufacturer.

### RNA Isolation, Sequencing, and Analysis

RNA was isolated from individual WT and MET KO mice liver samples at 0, 6, and 24 hours (*n* = 3) using the TRIzol method. The RNA samples were submitted to Novogene (Sacramento, CA) for quality verification, library preparation, RNA sequencing, and alignment to the mouse reference genome mm39 using the STAR program. The raw and processed RNA-sequencing data were submitted to the Gene Expression Omnibus database (*http://www.ncbi.nlm.nih.gov/geo*; accession number GSE305543). Differentially expressed genes (DEGs) (*P* < 0.05) were further filtered by using a fold change criterion (1.5-fold; either up-regulated or down-regulated) before downstream analysis. Both temporal (6 and 24 hours vs 0 hours) and intergroup (KO vs WT at specific time points) analyses were conducted. Based on observed changes in downstream gene expression patterns, altered canonical signaling pathways and upstream regulators were predicted using Ingenuity Pathways Analysis (IPA) version 8.0 (Ingenuity Systems, Redwood, CA). Enriched biological processes (Gene Ontology terms), Kyoto Encyclopedia of Genes and Genomes pathways, and Reactome pathways were identified using Database for Annotation, Visualization, and Integrated Discovery analysis software (DAVID Knowledgebase version 2021q4; *https://davidbioinformatics.nih.gov*), with comparisons made to the *Mus musculus* reference gene list. The enrichment plot was generated by using Gene Set Enrichment Analysis (GSEA version 4.4.4, build 16). GSEA was performed independently for the 6- and 24-hour timepoints, applying the gene-set permutation type. The analysis used the m5.go.bp.v2024.1.Mm.symbols.gmt and m2.cp.reactome.v2024.1.Mm.symbols.gmt database with the Mouse_Ensembl_Gene _ID_MSigDB.v2024.1.Mm.chip platform.

Publicly available spatial transcriptomic and single-nuclei RNA sequencing data sets (for hepatocytes) on human APAP-induced ALF and healthy livers were obtained from the Gene Expression Omnibus database (*http://www.ncbi.nlm.nih.gov/geo*; accession numbers GSE223559 and GSE223581, respectively).^21^ To identify genes relevant to APAP-induced ALF, we focused on those exhibiting a ≥2-fold change in addition to statistical significance. Furthermore, an independent human gene expression data set (*http://www.ncbi.nlm.nih.gov/geo*; accession number GSE120652) was also retrieved from the Gene Expression Omnibus database, comprising samples from three patients with APAP-induced ALF and three healthy control subjects.^22^ Genes exhibiting a significance threshold of *P* < 0.05 were selected for further analysis. DEGs identified from these data sets were further analyzed using IPA and DAVID tools to explore associated biological pathways.

### Statistical Analysis

The data are expressed as means ± SEM, and statistical comparisons were performed using a *t*-test with GraphPad Prism version 10.0 software (GraphPad Software, La Jolla, CA). Differences were considered statistically significant at *P* < 0.05.

## Results

### Aggravated Liver Injury in Hepatocyte-Specific MET KO Mice after APAP Overdose

**Figure 1:**
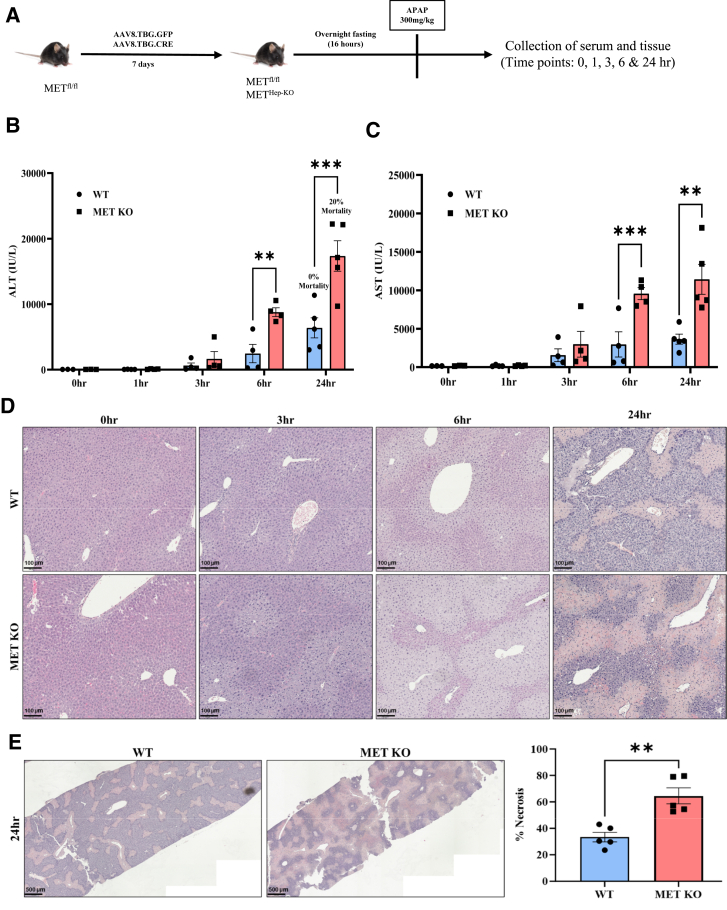
Aggravated liver injury in hepatocyte-specific MET knockout (KO) mice after acetaminophen (APAP) overdose. **A:** Schematics representing the experimental design in hepatocyte-specific MET KO mice. **B** and **C:** Bar graphs showing serum alanine aminotransferase (ALT) (**B**) and aspartate aminotransferase (AST) (**C**) levels at various timepoints. **D:** Representative photomicrographs of hematoxylin and eosin–stained liver sections displaying necrotic areas at various time points. **E:** Hematoxylin and eosin–stained sections displaying lower magnification. For all experiments, wild-type (WT) and MET KO mice were treated with 300 mg/kg APAP. Data are expressed as means ± SEM. *n* = 3 to 5. ∗∗*P* < 0.01 and ∗∗∗*P* < 0.001 versus WT mice. Scale bars: 100 μm (**D**); 500 μm [necrotic areas at 24 hours (**left panel**), with corresponding quantification of percentage necrosis (**right panel**)] (**E**).

In a previous study, a dose-dependent activation of MET signaling was observed after APAP overdose in mice.^11^ This study investigated whether MET plays a causal role in AILI using a hepatocyte-specific KO model, with the study design depicted in Figure 1A. The time-course studies were performed to cover both early liver injury and peak injury after APAP overdose.^23^^,^^24^ WT and MET KO mice showed no apparent histologic differences at baseline, along with normal serum alanine aminotransferase/aspartate aminotransferase levels (Figure 1, B–D). As expected, APAP-treated WT mice displayed extensive centrilobular necrosis and elevated serum alanine aminotransferase/aspartate aminotransferase levels with observable necrosis at 6 hours and injury peaking at 24 hours’ post-APAP (Figure 1, B–E). MET KO mice exhibited significantly higher alanine aminotransferase/aspartate aminotransferase levels (Figure 1, B and C) consistently at timepoints representing both early (6 hours) and peak (24 hours) injury, with around twofold higher necrotic area at 24 hours compared with WT mice (Figure 1, D and E). Extensive necrosis was linked to poor survival, with a 20% mortality rate in MET KO mice at 24 hours, whereas there was no mortality in WT mice (Figure 1B). Due to significant mortality in MET KO mice, later timepoints could not be pursued.

### Effects of MET Ablation on APAP-Protein Adducts Formation, GSH Levels, and Antioxidant Response

**Figure 2:**
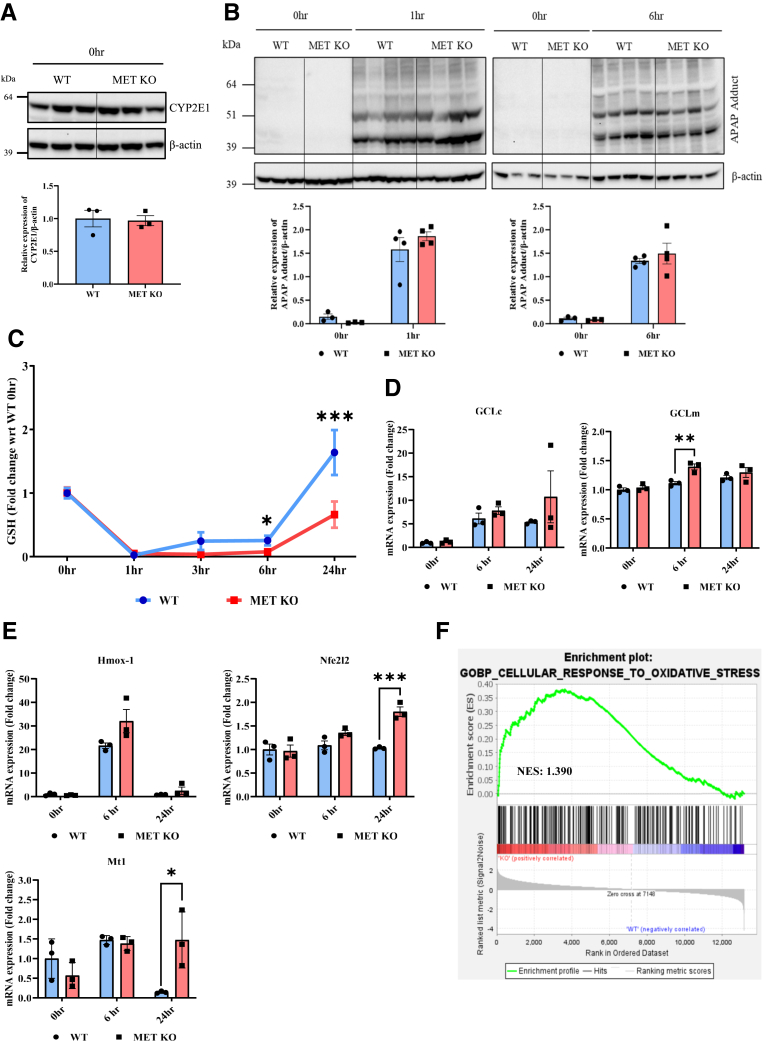
Effects of MET ablation on acetaminophen (APAP)-protein adducts formation, glutathione levels, and antioxidant genes. **A** and **B:** Immunoblot images and densitometric analysis representing the expressions of cytochrome P450 2E1 (CYP2E1) at basal level (0 hours) (**A**) and APAP-protein adducts at 1 and 6 hours’ post-APAP administration (**B**) in total liver lysates. **C:** Glutathione (GSH) levels at different timepoints. **D** and **E:** Bar graphs representing mRNA expression of glutamate-cysteine ligase catalytic subunit (GCLc) and modifier subunit (GCLm) (**D**) and Hmox-1, Nfe2l2, and Mt1 (**E**). **F:** Gene Set Enrichment Analysis plot showing enriched “cellular responses to oxidative stress” biological process in MET KO mice at 6 hours. For all experiments, wild type (WT) and MET knockout (KO) mice were treated with 300 mg/kg APAP. Data are expressed as means ± SEM. *n* = 3 to 5. ∗*P* < 0.05, ∗∗*P* < 0.01, and ∗∗∗*P* < 0.001 versus WT mice. NES, normalized enrichment score; wrt, with respect to.

To further elucidate the mechanisms underlying aggravated APAP hepatotoxicity in MET KO mice, several key processes involved in the initiation and progression of AILI were investigated. The initial phase of APAP hepatotoxicity involves formation of the reactive metabolite NAPQI, which rapidly conjugates with GSH, leading to its depletion. The excessive accumulation of NAPQI results in its binding to cellular proteins, particularly mitochondrial proteins, which leads to the formation of APAP-protein adducts.^23^^,^^25^ MET deletion did not affect cytochrome P450 2E1 expression, the major enzyme that metabolizes APAP to NAPQI at the basal level (0 hours) (Figure 2A) or at 1 and 6 hours after APAP overdose (Supplemental Figure S2, A and B). Furthermore, the initial APAP-protein adduct formation (at 1 hour) was similar in both WT and MET KO mice, indicating comparable metabolic activation of APAP to NAPQI in these groups (Figure 2B). APAP-protein adduct levels were also comparable in both groups at 6 hours (Figure 2B).

Furthermore, the initial depletion of GSH (at 1 hour), considered another surrogate marker of APAP metabolic activation,^23^^,^^24^ was similar in WT and MET KO mice (Figure 2C). GSH is resynthesized over time after initial depletion following APAP overdose, which is an important compensatory response for limiting progression of oxidative stress and associated liver injury.^23^ Interestingly, the replenishment of GSH (at 24 hours) was significantly reduced in MET KO mice (Figure 2C). Glutamate-cysteine ligase (GCL) is the rate-limiting enzyme involved in GSH synthesis and is composed of two subunits, GCLc (catalytic subunit) and GCLm (modifier subunit). GCLc is especially important because its induction after APAP overdose is critical for GSH synthesis.^23^ The induction of GCLc mRNA expression was not altered in MET KO mice, whereas GCLm mRNA expression was slightly higher (at 6 hours) in MET KO mice (Figure 2D). However, immunoblot analysis revealed no significant differences in GCLc or GCLm protein expression at either 6 or 24 hours (Supplemental Figure S2, C and D). Similarly, expressions of several antioxidant genes, including *Hmox-1*, the transcription factor Nrf2 (encoded by *Nfe2l2* gene), and nonenzymatic antioxidant *Mt1*, were either not affected or were higher in the MET KO group (Figure 2E). Overall, induction of antioxidant genes was not impaired in MET KO mice and, in fact, an enhanced cellular response to reactive oxygen species (as evidenced by the GSEA plot based on changes in the global gene expression profile using RNA sequencing) (Figure 2F) was observed in MET KO mice; this is most likely a compensatory response to higher oxidative stress and liver damage.

### MET Deletion Enhances JNK Activation via Inhibiting AKT Signaling, Aggravating AILI

**Figure 3:**
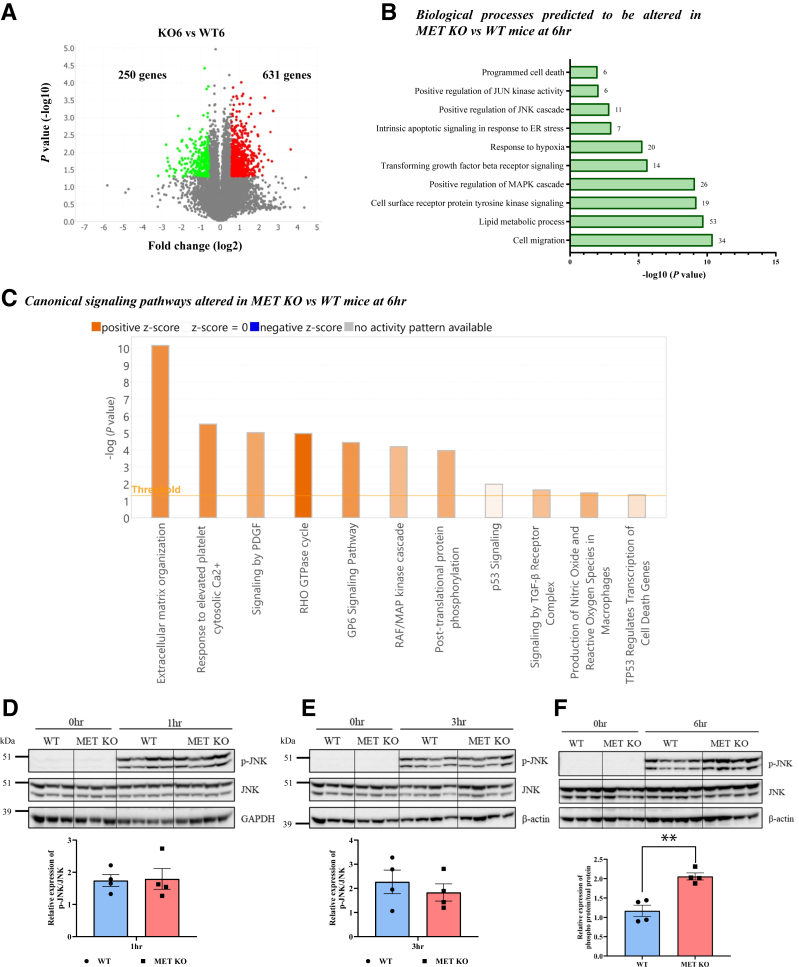
MET deletion enhances c-Jun N-terminal kinase (JNK) activation during acetaminophen (APAP)-induced liver injury. **A:** Volcano plot representing up and down-regulated genes at 6 hours in MET knockout (KO) mice versus wild-type (WT) mice. **B:** Enrichment analysis using DAVID analysis software showing altered biological processes (Gene Ontology terms). The number of differentially expressed genes associated with each pathway is indicated on the right side of the corresponding bar. **C:** Ingenuity Pathway Analysis showing altered canonical signaling pathways in MET KO versus WT mice at 6 hours (positive *z* score indicates predicted activation of a pathway). JNK/mitogen-activated protein kinase (MAPK) pathways were altered, among others. **D–F:** Immunoblot images and densitometric analysis representing the expressions of phospho-JNK (p-JNK) (T183/Y185) and JNK at 1 (**D**), 3 (**E**), and 6 (**F**) hours after APAP administration. For all experiments, WT and MET KO mice were treated with 300 mg/kg APAP. Data are expressed as means ± SEM. *n* = 3 to 4. ∗∗*P* < 0.01 versus WT mice. ER, endoplasmic reticulum; GAPDH, glyceraldehyde-3-phosphate dehydrogenase; PDGF, platelet-derived growth factor; TGF-β, transforming growth factor-β.

To further understand the mechanisms underlying aggravated early liver injury in MET KO mice, an unbiased pathway analysis was performed on DEGs obtained from RNA-sequencing in MET KO mice versus WT mice at 6 hours (831 DEGs: 631 up-regulated, 250 down-regulated) (Figure 3A). Mitogen-activated protein kinase/JNK signaling was among the top-most activated/altered pathways consistently in both DAVID analysis and IPA (Figure 3, B and C). It is well established that phosphorylation-mediated activation and mitochondrial translocation of JNK plays an important role in amplifying mitochondrial oxidative damage and is essential for necrotic cell death during AILI.^26^^,^^27^ Corroborating transcriptomic analysis, enhanced JNK phosphorylation (ie, activation) was observed in MET KO mice at 6 hours (Figure 3F). There was no observable difference in JNK activation between WT and MET KO mice at very early timepoints (1 and 3 hours) (Figure 3, D and E). Earlier studies have reported that stress-induced activation of JNK is negatively regulated by AKT.^28^, ^29^, ^30^, ^31^

**Figure 4:**
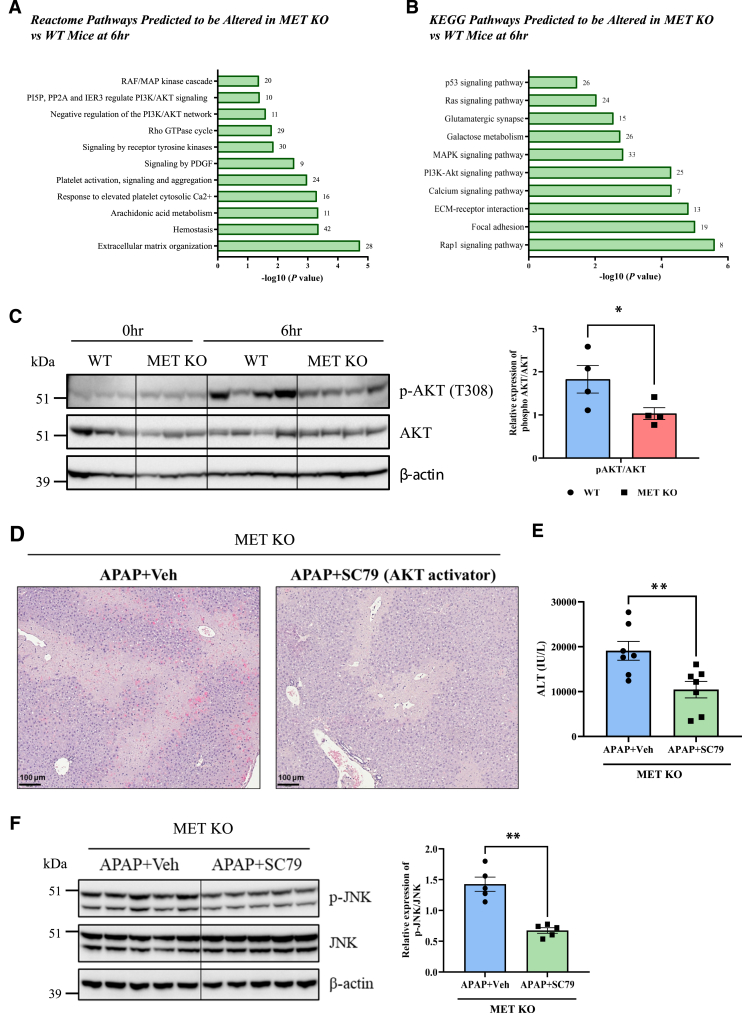
MET deletion inhibits AKT signaling enhancing c-Jun N-terminal kinase (JNK) activation and acetaminophen (APAP)-induced liver injury. **A** and **B:** Enrichment analysis using DAVID analysis software showing modulation of phosphatidylinositol 3-kinase (PI3K)/AKT signaling, among other altered Reactome (**A**) and Kyoto Encyclopedia of Genes and Genomes (KEGG) (**B**) pathways in MET knockout (KO) versus wild-type (WT) mice at 6 hours after APAP administration. The number of differentially expressed genes associated with each pathway is indicated on the right side of the corresponding bar. **C:** Immunoblot images and densitometric analysis showing expressions of phospho-AKT (p-AKT) (T308) and AKT at 6 hours. **D** and **E:** Representative photomicrographs of hematoxylin and eosin–stained liver sections displaying necrotic areas at 24 hours (**D**) and corresponding serum alanine aminotransferase (ALT) levels (**E**) in MET KO mice treated with APAP alone and in combination with SC79 (AKT activator). **F:** Immunoblot images and densitometric analysis representing the expressions of phospho-JNK (p-JNK) (T183/Y185) and JNK in total liver lysates at 6 hours in MET KO mice treated with APAP alone and in combination with SC79 (AKT activator). AKT activator (SC79: 10 mg/kg) or its vehicle (4% dimethyl sulfoxide) was administered 2 hours’ post-APAP overdose in MET KO mice, and liver tissue samples were collected at 6 and 24 hours. Data are expressed as means ± SEM. *n* = 3 to 4 (**C**); *n* = 5 to 7 (**E** and **F**). ∗*P* < 0.05 versus WT mice. ∗∗*P* < 0.01 versus vehicle (Veh)-control MET KO mice. Scale bar: 100 μm (**D**). ECM, extracellular matrix; MAPK, mitogen-activated protein kinase; PDGF, platelet-derived growth factor.

Apart from mitogen-activated protein kinase/JNK signaling, phosphatidylinositol 3-kinase/AKT signaling was also significantly altered in MET KO mice based on Reactome and Kyoto Encyclopedia of Genes and Genomes pathway analysis of the transcriptomic data at 6 hours’ post-APAP (Figure 4, A and B), which was further validated using immunoblotting (Figure 4C). AKT phosphorylation was markedly increased in WT mice after APAP treatment (6 hours vs 0 hours), and deletion of MET significantly decreased AKT phosphorylation (Figure 4C). To further explore whether MET regulates JNK activation and AILI via AKT signaling, an AKT activator (SC79) was used in MET KO mice. Notably, treatment with SC79 reduced JNK activation and liver injury following APAP overdose in MET KO mice (Figure 4, D–F). Thus, these current findings indicate that MET deletion suppresses AKT signaling and in turn, enhances JNK activation, which is one of the mechanisms contributing to exacerbation of AILI in MET KO mice.

In addition to modulation of mitogen-activated protein kinase/JNK and phosphatidylinositol 3-kinase/AKT pathways, significant alterations in several other relevant biological pathways were observed in MET KO mice versus WT mice at 6 hours, including transforming growth factor (TGF)-β signaling and programmed cell death, as indicated by the IPA and DAVID analysis (Figure 3, B and C). Interestingly, TGF-β1 emerged as one of the most prominent upstream regulators, predicted to be activated in APAP-overdosed MET KO mice compared with WT mice, with an activation *z* score of 4.591 (Supplemental Figure S3A and Supplemental Table S1), and the gene expression of TGF-β3 was also significantly elevated at the 6-hour mark (Supplemental Figure S3B). TGF-β signaling has been reported not only to inhibit regeneration but also contribute to liver injury via JNK activation after APAP overdose.^32^ Lastly, increased gene expression of several cell death signaling mediators, including RIP kinases, Bax, Bbc3, Pmaip1, Apaf1, Aif1, and Thbs1, were observed in MET KO mice compared with WT mice (Supplemental Figure S3C).

### Deletion of MET Was Associated with Increased Mitochondrial Damage and Release of Apoptosis-Inducing Factor into Cytosol

During AILI, activated JNK translocates to mitochondria and amplifies mitochondrial oxidant damage and release of cell death mediators.^4^^,^^25^ The current study therefore investigated whether excess JNK activation upon MET deletion was accompanied with amplification of mitochondrial damage and associated cell death signaling.

**Figure 5:**
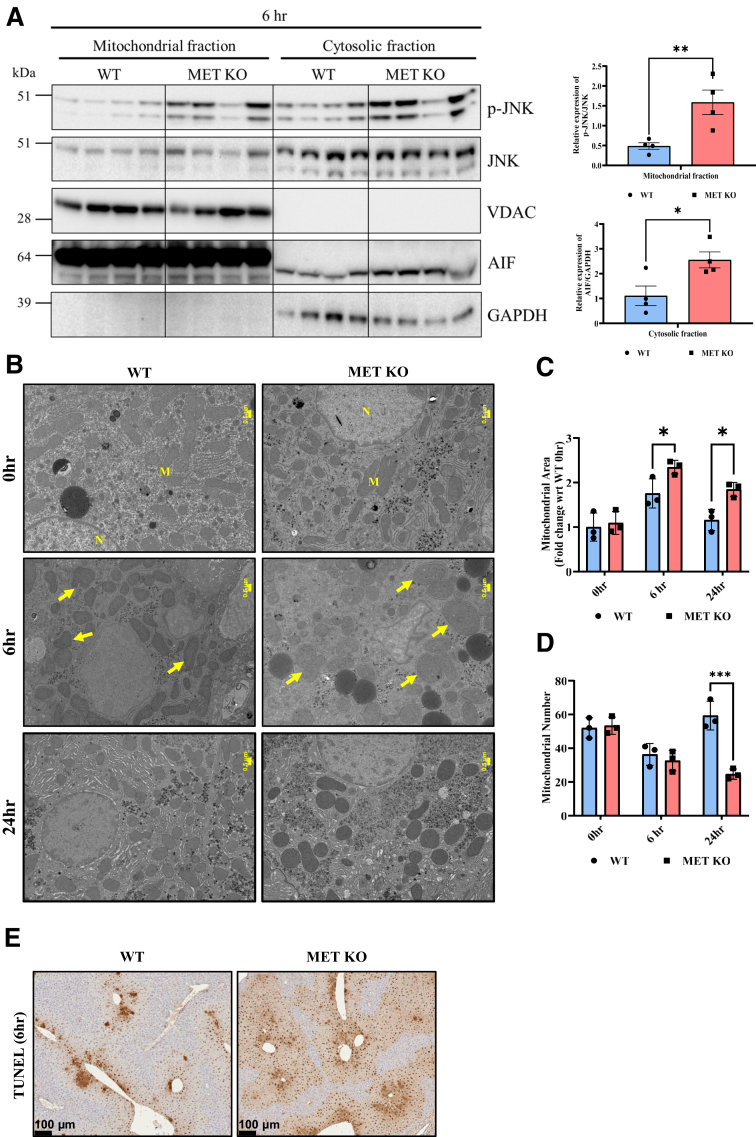
Deletion of MET was associated with increased mitochondrial damage and release of apoptosis-inducing factor (AIF) into cytosol. **A:** Immunoblot images and densitometric analysis representing the expressions of phospho–c-Jun N-terminal kinase (p-JNK) , JNK, and AIF in liver mitochondrial and cytosolic lysates at 6 hours after acetaminophen (APAP) administration. **B:** Representative photomicrographs of 70 nm liver sections depicting mitochondrial morphology using transmission electron microscopy imaging after 6 and 24 hours of APAP administration. **Yellow arrows** denote swollen mitochondria with partial loss of cristae. **C** and **D:** Bar graphs display mitochondrial area (**C**) and mitochondrial number (**D**). **E:** Representative photomicrographs of liver sections stained for detecting DNA fragmentation [terminal deoxynucleotidyl transferase-mediated dUTP nick-end labeling (TUNEL)] at 6 hours. For all experiments, MET knockout (KO) and wild-type (WT) mice were treated with 300 mg/kg APAP. Data are expressed as means ± SEM. (n = 3 to 4). ∗*P* < 0.05, ∗∗*P* < 0.01, and ∗∗∗*P* < 0.001 versus WT mice. Scale bars: 0.5 μm (**B**); 100 μm (**E**). GAPDH, glyceraldehyde-3-phosphate dehydrogenase; M, mitochondria; N, nucleus; VDAC, voltage-dependent anion channel; wrt, with respect to.

Consistent with higher JNK activation, increased mitochondrial levels of activated JNK were noted at 6 hours in MET KO mice (Figure 5A). The MET KO group also displayed exacerbated mitochondrial swelling with partial loss of cristae as observed in transmission electron microscopy imaging (Figure 5B), which was further associated with increased mitochondrial area at 6 hours (Figure 5C) and a reduction in mitochondrial numbers at 24 hours (Figure 5D). Extensive mitochondrial damage opens the mitochondrial permeability transition pore and releases endonucleases such as apoptosis-inducing factor into cytosol, ultimately leading to nuclear DNA damage and necrotic cell death in AILI.^33^ Consistently, MET KO mice exhibited an increased release of apoptosis-inducing factor into cytosol, further showing increased mitochondrial damage (Figure 5A); this was associated with increased DNA strand breaks as detected by terminal deoxynucleotidyl transferase-mediated dUTP nick-end labeling staining (Figure 5E).

### Impaired Compensatory Proliferative Response in MET KO Mice

**Figure 6:**
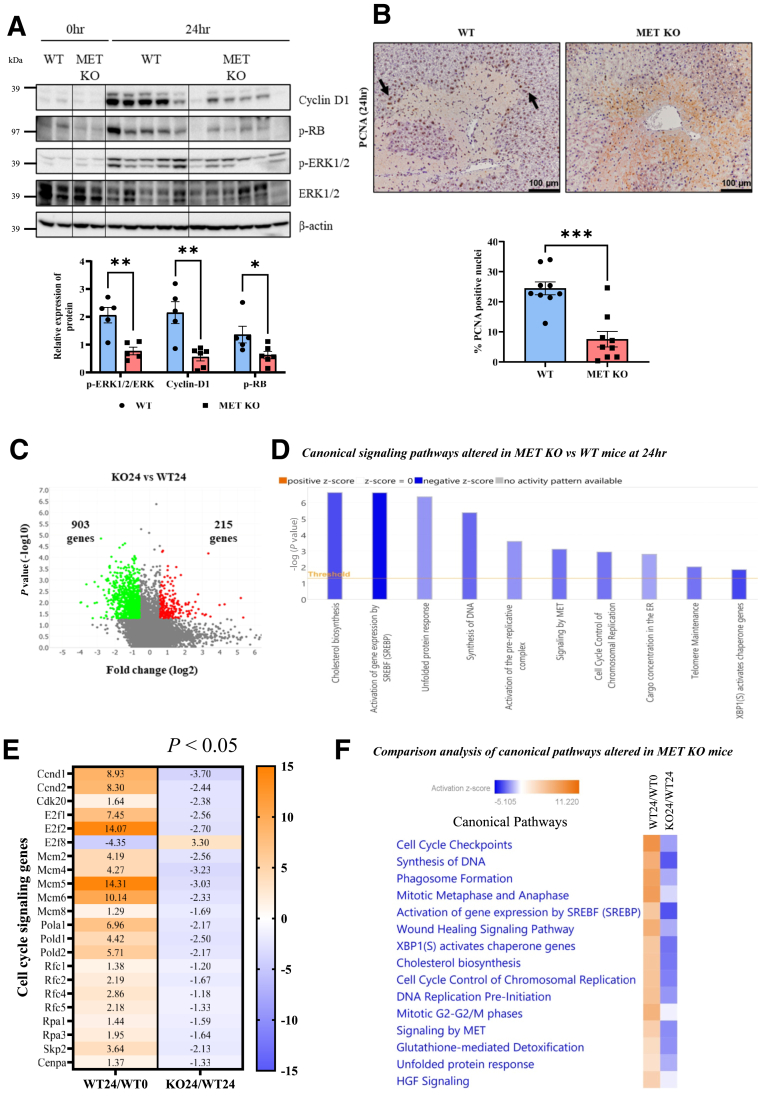
Impaired compensatory proliferative response in MET knockout (KO) mice. **A:** Immunoblot images and densitometric analysis representing the expressions of Cyclin-D1, phospho-RB, phospho–extracellular signal–regulated kinase (p-ERK) 1/2, and ERK1/2 in total liver lysates after 24 hours of acetaminophen administration. **B:** Representative photomicrographs of proliferating cell nuclear antigen (PCNA)-stained liver sections at 24 hours. **Black arrows** indicate cells with dark-brown nuclear PCNA staining surrounding necrotic zones showing proliferating hepatocytes in S-phase. For all experiments, mice were treated with 300 mg/kg acetaminophen. **C:** Volcano plot representing up-regulated and down-regulated genes at 24 hours in MET KO (KO24) versus wild type (WT24) mice. **D:** Altered canonical signaling pathways in MET KO versus WT mice at 24 hours, identified using Ingenuity Pathways Analysis (negative *z* score indicates predicted inhibition of a pathway). **E** and **F:** Heat maps showing comparison of cell cycle gene expression (**E**) and comparison of altered canonical pathways (**F**) in WT mice at 24 hours (vs 0 hours) and in MET KO mice at 24 hours (vs WT mice at 24 hours). Intensity of the blue color reflects extent of down-regulation/inhibition, and the orange color reflects up-regulation/activation of a particular gene or canonical pathway. Data are expressed as means ± SEM. ∗*P* < 0.05, ∗∗*P* < 0.01, and ∗∗∗*P* < 0.001 versus WT mice. *n* = 3 to 6 (**B**). Scale bar: 100 μm (**B**). ER, endoplasmic reticulum; HGF, hepatocyte growth factor.

Next, the impact of MET deletion on compensatory proliferative response after AILI was investigated. Deletion of MET from hepatocytes resulted in reduced induction of core cell cycle protein cyclin D1 and subsequent phosphorylation of RB protein that regulates entry into the cell cycle (Figure 6A). Furthermore, MET KO mice displayed reduced activation of extracellular signal–regulated kinase 1/2 (ERK1/2), the upstream proliferative signal that is known to regulate cell cycle (Figure 6A). Consistently, immunohistochemistry revealed notably decreased numbers of proliferating cell nuclear antigen–positive nuclei surrounding necrotic zones at 24 hours in MET KO mice compared with control mice, indicating impaired hepatocyte proliferation (Figure 6B). Overall, analysis of DEGs (total of 1118: 215 up-regulated, 903 down-regulated) (Figure 6C) in MET KO mice versus WT mice at 24 hours using IPA revealed cell cycle control of chromosomal replication and synthesis of DNA among the top canonical pathways inhibited by MET deletion (Figure 6D). Furthermore, several important markers associated with cell cycle progression and DNA synthesis, including Ccnd1, E2f1, E2f2, Mcm, and Rfc genes, were induced in WT mice but were markedly down-regulated in MET KO mice (Figure 6E). In fact, comparative analysis using IPA revealed that several of the canonical pathways related to proliferation, including DNA replication pre-initiation and mitotic metaphase/anaphase, were activated in WT mice upon APAP treatment but were inhibited in MET KO mice (Figure 6F).

In addition to IPA, DAVID analysis (Reactome pathways, Gene Ontology biological processes, and Kyoto Encyclopedia of Genes and Genomes pathways) also revealed significant alterations in cell cycle pathways in MET KO mice (Supplemental Figure S4, A–C). Moreover, GSEA showed robust inhibition of DNA replication in MET KO mice (Supplemental Figure S4D). Other affected pathways included DNA repair and damage bypass, response to oxidative stress, endoplasmic reticulum (ER) unfolded protein response (UPR), protein processing in the ER, and the ER-associated protein degradation pathway (Supplemental Figure S4, A–C).

Along with repression of proliferative signals, MET KO mice displayed enhanced levels of anti-proliferative proteins such as TGF-β1 and p21 (Supplemental Figure S4E), which have been previously shown to induce senescence and impair recovery after APAP exposure in mice and humans.^5^^,^^34^ Apart from TGF-β1 signaling, a recent study reported an important role of CXCL14 in regulating p21 in the peri-necrotic hepatocytes and impairing regeneration after severe APAP overdose in mice.^35^ CXCL14 was also identified as an early prognostic biomarker for poor outcomes in patients with APAP-induced ALF.^36^

Interestingly, in the current study, CXCL14 mRNA expression was consistently higher (around twofold) in MET KO mice versus WT mice at both 6 and 24 hours, with overall expression markedly higher at 24 hours compared with 6 hours (Supplemental Figure S4F). Although these changes did not attain statistical significance, CXCL14 expression did correlate with the severity of injury and impaired regeneration, very consistent with the earlier studies.^35^^,^^36^ MET deletion also affected other signals of repair apart from proliferation. For instance, Annexin A2 induction was drastically reduced at the 24-hour mark after APAP overdose in MET KO mice versus WT mice (Supplemental Figure S4G), which has recently been shown to be important for hepatocyte migration for wound closure following AILI in both mice and humans.^21^ Furthermore, poly (ADP-ribose) polymerase 1 induction, which is a crucial enzyme involved in the DNA repair process after APAP-driven DNA strand breakage, was reduced in MET KO mice at 24 hours (Supplemental Figure S4H).^37^

### Impact of Hepatocyte-Specific MET Deletion on UPR

**Figure 7:**
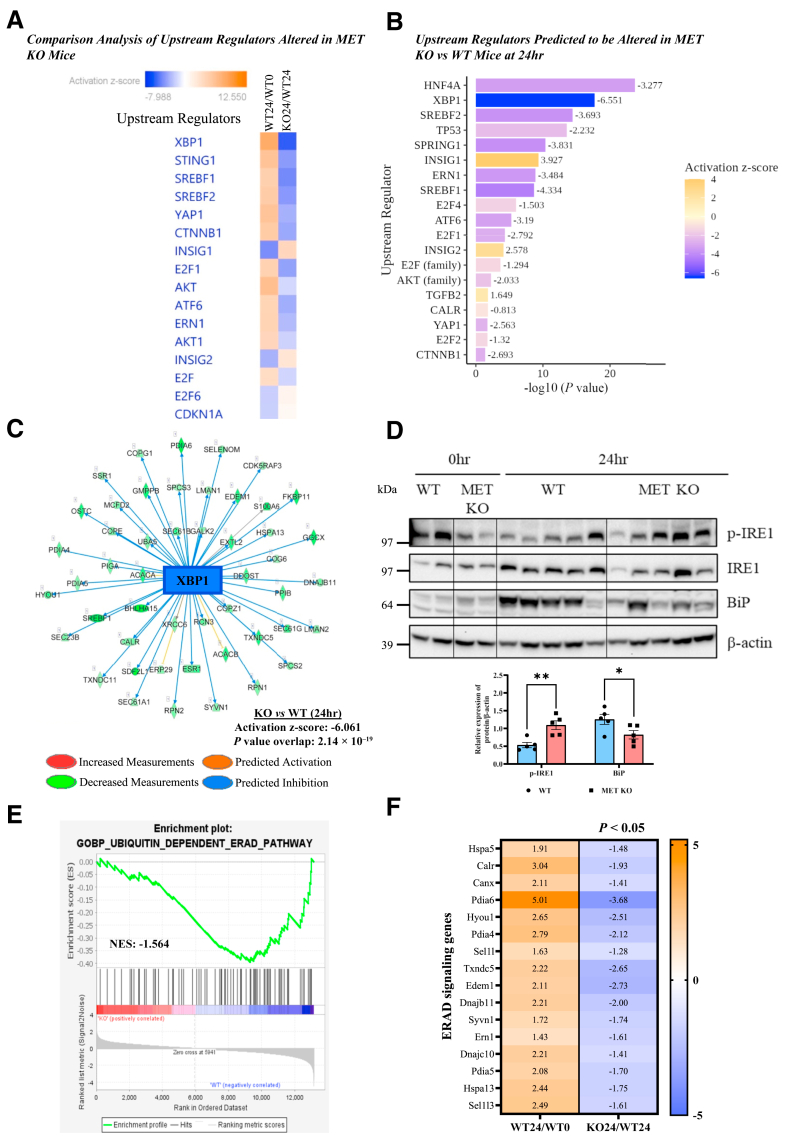
Impact of hepatocyte-specific MET deletion on unfolded-protein response. **A** and **F:** Heat maps depicting comparison analysis of altered upstream regulators (**A**) and comparison of endoplasmic reticulum–associated protein degradation (ERAD) gene (**F**) expression in wild type (WT) mice at 24 hours (WT24) [vs at 0 hours (WT0)] and in MET knockout (KO) mice at 24 hours (KO24) [vs WT mice at 24 hours (WT24)]. **B:** Upstream regulators predicted to be altered in MET KO mice versus WT mice at 24 hours identified by using Ingenuity Pathways Analysis. Intensity of the blue color reflects extent of inhibition, and the orange color reflects activation of a particular upstream regulator. **C:** Downstream gene network of XBP1 predicted to be significantly inhibited in MET KO mice after 24 hours of acetaminophen administration. **D:** Immunoblot images representing the expressions in total liver lysates of phospho–inositol-requiring enzyme 1 (p-IRE1) (S724), IRE1, and BiP at 24 hours. **E:** Gene Set Enrichment Analysis plot showing depleted “ubiquitin-dependent ERAD pathway” biological process in MET KO mice at 24 hours. For all experiments, mice were treated with 300 mg/kg acetaminophen. Data are expressed as means ± SEM. *n* = 3 to 5. ∗*P* < 0.05 and ∗∗*P* < 0.01 versus WT mice. NES, normalized enrichment score.

Along with proliferation signaling, UPR emerged among the top signals altered in hepatocyte-specific MET KO mice consistently in all pathway analyses of DEGs at 24 hours (Figure 6, D and F, and Supplemental Figure S4, A and B). APAP overdose is known to induce ER stress and X-box binding protein 1 splicing (ie, XBP1 activation).^38^ XBP1(s) (ie, spliced form) is an important transcriptional regulator of chaperone genes involved in proper folding/clearance of misfolded proteins after ER stress. XBP1 was predicted to be activated after APAP overdose in WT mice as expected but was inhibited in MET KO mice versus WT mice in comparative upstream regulator analysis (Figure 7A). In fact, XBP1 was among the topmost upstream regulators predicted to be significantly inhibited in APAP overdosed MET KO mice versus WT mice with an activation *z* score of –6.061 (Figure 7B). The inhibited downstream gene network of XBP1 is shown in Figure 7C and Supplemental Table S2.

Furthermore, IPA identified XBP1(s)-mediated activation of chaperone genes among the top canonical signaling pathways inhibited in APAP overdose MET KO mice versus WT mice (Figure 6D). This was additionally supported by markedly decreased induction of protein expression of the important chaperone BiP, a downstream target of XBP1(s), in MET KO mice (Figure 7D). Interestingly, inositol-requiring enzyme 1 activation, which causes XPB1 splicing and activation, remains higher in MET KO mice despite lower chaperone response (Figure 7D). Overall, the entire ER-associated protein degradation signaling pathway was repressed in MET KO mice, as shown by GSEA (Figure 7E). The suppressed ER-associated protein degradation cascade in the MET KO group is clearly shown by down-regulation of multiple genes [eg, *Hspa5* (encodes *Bip*), *Calr, Canx, Pdia4-a6, Hyou-1, Sel1l, Txndc5, Edem1, Dnajb11, Syvn1, Ern1, Txndc11, Dnajc10, Hspa13, Sel1l3*] responsible for the folding or clearance of misfolded proteins in the ER after UPR activation (Figure 7F).

Overall, these findings underscore the importance of MET in regulating UPR after AILI. In addition to maintaining ER homeostasis, XBP1s is involved in the regulation of synthesis and metabolism of lipids, and earlier studies have found that excessive APAP overdose in rodents can lead to increased hepatic triglyceride levels.^39^^,^^40^ MET KO mice exhibited decreased gene expression of key lipogenic genes (including *Srebf1, Acaca, Acacb, Fasn, Scd1,* and *Acyl*), which are crucial for fatty acid synthesis, after 24 hours of APAP treatment (Supplemental Figure S4I).

### HGF/MET Signaling Activation in Human ALF

**Figure 8:**
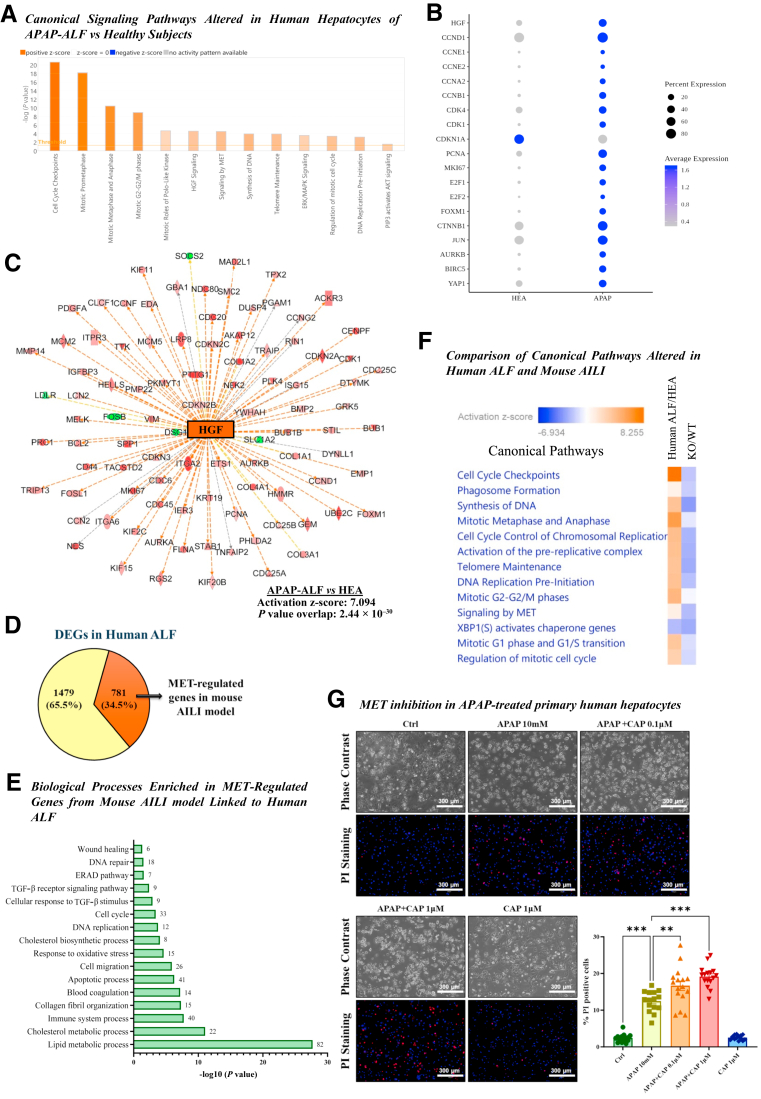
Hepatocyte growth factor (HGF)/MET signaling activation in human acute liver failure (ALF). **A:** Predicted alterations in canonical signaling pathways in human hepatocytes from acetaminophen (APAP)-induced ALF versus healthy livers identified by Ingenuity Pathways Analysis of publicly available single-nuclei RNA-sequencing data set (positive *z* score indicates predicted activation of a pathway). **B:** The dot plot illustrates average expression along with percentage of cells showing significant expression of cell proliferation genes in APAP-induced ALF livers versus healthy controls, based on analysis of publicly available spatial transcriptomics data set. **C:** Downstream gene network of HGF predicted to be activated in hepatocytes of APAP-induced ALF livers based on single-nuclei RNA-sequencing data analysis. **D:** Pie-chart illustrating that a large proportion (approximately 35%) of genes altered in human ALF were regulated by MET in the mouse APAP-induced liver injury (AILI) model [ie, differentially expressed genes (DEGs) in MET knockout (KO) vs wild-type (WT) mice]. **E:** Enrichment analysis using DAVID analysis software showing altered biological processes (Gene Ontology terms) in the MET-regulated genes from the mouse AILI model linked to human ALF (ie, 781 genes shown in orange color in **D**). The number of genes associated with each pathway is indicated on the right side of the corresponding bar. **F:** Heat map depicting comparison analysis of altered canonical pathway in human ALF (vs HEA) and MET KO (vs WT) mice. **G:** Effect of MET inhibition on APAP-treated primary human hepatocytes. Representative images of propidium iodide (PI)-stained primary human hepatocytes, illustrating the percentage of cell death after 24 hours of treatment with 10 mM APAP alone or in combination with capmatinib (CAP) (MET inhibitor: 0.1 and 1 μM). ∗∗*P* < 0.01 and ∗∗∗*P* < 0.001 versus APAP 10 mM. Scale bar: 300 μm (**G**). ERAD, endoplasmic reticulum–associated protein degradation; HEA, healthy human liver tissue; TGF-β, transforming growth factor-β.

Next, publicly available data sets of single-nuclei RNA sequencing and spatial transcriptomics on human APAP-induced ALF livers were analyzed.^21^ These APAP-induced ALF livers were previously reported to show strong proliferation signals indicative of a regenerative response.^21^ Consistent with the published study,^21^ the current analysis showed strong activation of proliferation and cell cycle pathways/genes in these APAP-induced ALF human livers (Figure 8, A and B and Supplemental Figure S5, B–D). Interestingly, analysis of the spatial transcriptomics data revealed strong induction of HGF in APAP-induced ALF livers, specifically in the areas surrounding the necrotic zones, with an approximately 2.5-fold increase in percent cell expressing HGF and an approximately sixfold increase in overall HGF expression (Figure 8B and Supplemental Figure S5A).

Furthermore, upstream regulator analysis (using IPA) of the single-nuclei RNA sequencing data revealed striking activation of HGF downstream signaling in hepatocytes of human APAP-induced ALF livers compared with uninjured human liver with an activation *z* score of 7.094 (*P* value of overlap: 2.44E-30) (Figure 8C and Supplemental Figure S5D). Similar to the mouse AILI model, AKT and extracellular signal–regulated kinase signaling pathways were also activated in human APAP-induced ALF livers (Figure 8A). Analysis of another published transcriptomic data set also revealed activation of HGF/MET signaling along with proliferative signaling in human APAP-induced ALF (Supplemental Figure S6).^22^ Interestingly, correlation of this human APAP-induced ALF data set with the murine MET KO data set revealed that of 2714 significantly altered genes in human APAP-induced ALF, 34.5% (781 genes) showed differential regulation by MET in mouse AILI (ie, altered in MET KO mice compared with WT mice) (Figure 8D). This indicates that MET potentially regulates a significant proportion of genes relevant for human APAP-induced ALF. These MET-regulated genes in mouse AILI correlating with human ALF were enriched in relevant biological processes such as cell cycle, cell death signaling, ER-associated protein degradation pathway, and TGF-β signaling (Figure 8E).

Lastly, a comparative analysis between the human and mouse data sets using IPA revealed that the key canonical signaling pathways associated with cell cycle progression, including DNA synthesis and mitosis, were strongly activated in human APAP-induced ALF versus healthy subjects (Figure 8F). In contrast, many of these pathways were strikingly inhibited in MET KO mice versus WT mice, indicating that MET potentially drives several signaling changes relevant for human APAP-induced ALF (Figure 8F).

### MET Inhibition Aggravates APAP-Induced Cell Death in Primary Human Hepatocytes

APAP-induced hepatocyte death has been previously modeled and well characterized in freshly isolated primary human hepatocytes.^41^ Thus, to bolster the human relevance of current findings, the role of MET in regulating APAP-induced cell death was investigated in freshly isolated primary human hepatocytes. As expected, APAP treatment increased cell death (as measured by propidium iodide staining) at 24 hours in primary human hepatocytes (Figure 8G). MET inhibition (without APAP) did not increase cell death, indicating MET inhibitor *per se* was nontoxic to primary human hepatocytes at the concentrations used in this study. Importantly, MET inhibition aggravated cell death in APAP-treated primary human hepatocytes in a concentration-dependent manner, showing the role of MET in restricting APAP-induced hepatocyte death in a human AILI model (Figure 8G).

## Discussion

APAP is widely used as an analgesic and antipyretic medication, but its accidental or intentional overdose is a major contributor to drug-induced liver injury. APAP overdose can result in acute liver injury or, in severe cases, ALF and mortality. A robust liver regeneration response is crucial for recovery and determining final outcome after APAP-induced ALF.^3^^,^^5^ HGF/MET signaling is known to promote hepatocyte proliferation and liver regeneration after PHx.^7^ Unlike PHx in a normal healthy liver, AILI is complicated by the presence of massive liver injury and inflammation that intricately governs the regenerative response. Despite its recognized significance in liver regeneration, the role of MET in AILI remains unexplored. The current study investigated the impact of hepatocyte-specific MET deletion on liver injury and compensatory proliferative response in a clinically relevant model of APAP overdose in mice.

Our earlier study had found dose-dependent activation of MET after APAP overdose in mice.^11^ Notably, MET activation occurred very early, even before any observable necrosis, indicating its potential role in liver injury, apart from liver regeneration. Indeed, the current study found that deletion of MET exacerbates liver injury very early and ultimately leads to a greater extent of total hepatic damage and mortality after APAP overdose in mice. Thus, the current study showed a protective role of MET in restricting liver injury and promoting survival after APAP overdose.

The onset of AILI commences with the generation of the reactive APAP metabolite NAPQI, which depletes cellular GSH and forms adducts with mitochondrial proteins initiating mitochondrial dysfunction/oxidative stress.^4^ This initial oxidative stress leads to JNK activation and its translocation to the mitochondria, which in turn further exacerbates mitochondrial oxidative stress, leading to a vicious loop of progressive JNK activation and mitochondrial oxidative damage. How this feed-forward loop of JNK activation is regulated remains unclear, however.

The current study showed that MET deletion enhances JNK activation and its mitochondrial translocation, leading to excessive mitochondrial damage and subsequent release of cell death factor, apoptosis-inducing factor, into cytosol, despite no change in initial APAP metabolic activation. Furthermore, this study revealed that the higher JNK activation in MET KO mice is most likely driven by reduced AKT activation in the absence of MET signaling, which is consistent with previous studies describing the role of AKT in repressing stress-induced JNK activation in other models.^28^, ^29^, ^30^ Treatment with an AKT activator reduced JNK activation and liver injury in MET KO mice, confirming the role of the AKT/JNK pathway in the phenotype observed in MET KO mice. It is important to note that JNK activation was increased at 6 hours’ post-APAP in MET KO mice, but early JNK activation (1 or 3 hours’ post-APAP) was not altered. Furthermore, an AKT inhibitor was administered at 2 hours, when JNK was already activated, but it still decreased prolonged JNK activation and liver injury at later timepoints in MET KO mice. These data support the hypothesis that continuous JNK activation is important to maintain the vicious loop of oxidative damage during AILI; MET plays an important role in limiting this progressive oxidative damage and JNK activation but does not affect initial JNK activation. Thus, the current study indicates that MET activation and its inhibitory effect on JNK via AKT signaling is an important survival response aimed to restrict liver injury after APAP overdose.

Apart from aggravating liver injury, hepatocyte-specific deletion of MET dramatically reduced the compensatory hepatocyte proliferation after APAP overdose. This action was associated with diminished activation of extracellular signal–regulated kinase signaling, leading to impaired cell cycle activation with reduced levels of a core cell cycle initiator, cyclin-D1, along with induction of cell cycle inhibitors such as p21. Considering that liver injury was higher in MET KO mice, and severe liver injury is known to impair compensatory regenerative response in the AILI model,^11^ it is likely that the exacerbated liver injury in MET KO mice contributed to the lower hepatocyte proliferation observed in these mice. Thus, a direct role of MET in liver regeneration after APAP overdose could not be established using the MET deletion strategy used in this study. Further studies are required to show any direct role of MET in liver regeneration in the AILI model by using strategies that do not alter initial injury, such as pharmacologic MET inhibition post-APAP treatment. Nevertheless, indirectly or directly, the current study indicates that MET modulates the compensatory proliferative response.

From a therapeutic standpoint, one open question is whether stimulation of MET signaling can be a viable approach to restrict progression of injury and/or improve regeneration. In our earlier study, MET was activated after a moderately toxic dose of APAP that was associated with a robust liver regeneration response.^11^ However, MET activation was even higher (but still transient) after a severely toxic APAP overdose, in which liver regeneration was impaired. This indicates that although MET activation might be an important survival response, it may not be sufficient alone to induce repair in the failing livers after severe injury. This is also supported by an earlier study in which serum HGF levels were higher in nonsurvivors compared with survivors in patients with APAP-induced ALF.^42^ In contrast, several recent studies indicate that HGF interventions even after severe AILI can decrease injury, improve proliferation/resolution of injury, or enhance survival in the murine model.^43^, ^44^, ^45^ Further comprehensive temporal studies are required to investigate if late and prolonged MET activation via HGF either alone or in combination with other factors can decrease liver injury and/or improve liver regeneration/repair, altering the final outcome of AILI.

Comparative and temporal transcriptomic analysis revealed that pathways related to DNA synthesis, cell cycle progression, and proliferation failed to be activated in MET KO mice during the regenerative phase; in addition, pathways related to cell death signaling and senescence were activated in MET KO mice very early on. These include activation of the TGF-β signaling pathway, which has been previously shown to not only induce senescence and impair recovery/regeneration but also directly contribute to liver injury via JNK activation after APAP overdose.^5^^,^^32^^,^^34^ Apart from these pathways, XBP1 and UPR were consistently found to be among the top significant pathway/upstream regulators inhibited in MET KO mice in all the transcriptomic analyses. XBP1 is typically activated in response to cellular stress as a part of the cell’s adaptive mechanisms to induce chaperones involved in folding or clearance of misfolded proteins to restore ER homeostasis. Strikingly, several chaperones that are induced after APAP overdose were repressed in MET KO mice. The impaired UPR further indicates diminished repair capacity in MET KO mice after APAP overdose.

The human relevance of the current findings was shown by analysis of previously published spatial transcriptomics data sets of livers from patients with APAP-induced ALF.^21^ These liver samples were previously reported to exhibit strong proliferation signals indicative of a regenerative response.^21^ Our analysis of these data sets revealed that HGF was specifically induced in areas surrounding the necrotic zones in these livers. Further analysis of the published single-nuclei RNA-sequencing data of these livers showed that robust stimulation of multiple proliferative signaling pathways was accompanied by strong activation of HGF/MET signaling in hepatocytes.^21^ It should be noted that in the majority of the APAP-induced ALF cases, the liver regenerates spontaneously, resulting in survival, and only very severe ALF cases result in failed recovery and mortality.^1^ However, no association between survival and MET signaling activation was possible to deduce based on these data sets. Nevertheless, our analysis does indicate that a strong proliferative response in the liver of these patients with APAP-ALF was associated with HGF/MET signaling activation. Interestingly, 35% of the genes altered in human APAP-induced ALF were found to be regulated by MET based on murine MET KO study, and these genes were enriched for biological processes associated not only with proliferation but also with senescence and cell death signaling.^22^ This indicates that MET potentially drives a substantial fraction of gene expression changes relevant for human APAP-induced ALF. Finally, MET inhibition in APAP-treated primary human hepatocytes increased cell death, showing a protective role of MET in restricting APAP-induced hepatocyte damage in a human model. Overall, all of these data indicate that MET activation might be an important survival response even in human APAP-induced ALF.

In conclusion, the current study uncovered a role of MET in restricting liver damage after APAP overdose in mice using a hepatocyte-specific deletion strategy. Deletion of MET exacerbated initial liver injury by promoting mitochondrial damage and associated cell death signaling pathways and also impaired subsequent hepatocyte proliferative response. The effects on liver injury were at least partly driven by the inhibition of AKT signaling upon MET ablation, resulting in enhanced JNK activation. The human relevance of MET signaling for APAP-induced ALF was also shown. Overall, this study indicates an important role of MET signaling in limiting liver injury driving a critical survival response after APAP-induced ALF.

## Disclosure Statement

None declared.

## Supplemental Data

Supplemental Figure S1 Quantification of MET knock-down by AAV8.TBG.PI.Cre.rBG. **A:** Representative Sashimi plot of RNA-sequencing libraries from MET wild type (WT) and knockout (KO) livers after 6 hours of treatment. The number of intron-spanning reads is marked above the arc that links exon splice sites. The **dotted lines** mark the position of exon 16, which is deleted in the KO model. The in-frame deletion removes a critical ATP-binding site in the intracellular tyrosine kinase domain, essential for the activation of MET signaling. **B:** Fraction of spanning reads that join exon 15 to 17, averaged from three livers in each experimental group. ∗∗∗*P* < 0.001 versus WT mice.

Supplemental Figure S2 **A–D:** Immunoblot images and densitometric analysis representing the expressions of cytochrome P450 2E1 (CYP2E1) at 1 hour (**A**) and 6 hours (**B**) and glutamate-cysteine ligase catalytic subunit (GCLc) and modifier subunit (GCLm) at 6 hours (**C**) and 24 hours (**D**) after acetaminophen treatment in wild-type (WT) and MET knockout (KO) mice.

Supplemental Figure S3 **A:** Upstream regulators predicted to be altered in MET knockout (KO) versus wild-type (WT) mice at 6 hours identified by using Ingenuity Pathway Analysis. Intensity of the blue color reflects extent of inhibition, and the orange color reflects up-regulation of a particular upstream regulator. **B:** Bar graph representing mRNA expression of transforming growth factor-β3 (TGF-β3). **C:** Bubble plot representing up-regulation of cell death-associated genes in MET KO versus WT mice at 6 hours (intensity of green color indicates *P* value significance).∗*P* < 0.05 versus WT mice.

Supplemental Figure S4 **A–C:** Enrichment analysis using DAVID analysis software showing altered biological processes (Gene Ontology terms) (**A**), Kyoto Encyclopedia of Genes and Genomes (KEGG) pathways (**B**), and Reactome pathways (**C**) in MET knockout (KO) versus wild-type (WT) mice at 24 hours after acetaminophen administration. The number of differentially expressed genes associated with each pathway is indicated on the right side of the corresponding bar. **D:** Gene Set Enrichment Analysis plot showing depleted “DNA replication” Reactome pathway in MET KO mice versus WT mice at 24 hours. **E:** Immunoblot images representing the expressions of transforming growth factor-β1 (TGF-β1) and p21 in total liver lysates at 24 hours. **F–H:** Bar graphs representing mRNA expression of CXCL14 (**F**), Anxa2 (**G**), and poly (ADP-ribose) polymerase 1 (PARP1) (**H**). **I:** Bubble plot showing the down-regulation of lipogenic genes in MET KO mice at 24 hours (the intensity of the red color indicates *P* value significance). ∗*P* < 0.05 and ∗∗∗*P* < 0.001 versus WT mice. ER, endoplasmic reticulum; ERAD, endoplasmic reticulum–associated protein degradation; NES, normalized enrichment score.

Supplemental Figure S5 **A:** Analysis of publicly available spatial transcriptomics data set (Gene Expression Omnibus database, *http://www.ncbi.nlm.nih.gov/geo*; accession number GSE223559) showing increased expression of hepatocyte growth factor (HGF) localized around necrotic areas in human acetaminophen (APAP)-induced acute liver failure (ALF) livers. **B** and **C:** Enrichment analysis of publicly available single-nuclei RNA sequencing data set (Gene Expression Omnibus database, *http://www.ncbi.nlm.nih.gov/geo*; accession number GSE223581) using DAVID analysis software showing biological processes (Gene Ontology terms) (**B**) and Reactome pathways (**C**) predicted to be altered in human hepatocytes of APAP-ALF versus healthy subjects. The number of genes associated with each pathway is indicated on the right side of the corresponding bar. **D:** Upstream regulators predicted to be altered in hepatocytes of APAP-ALF patients. Intensity of the blue color reflects extent of inhibition, and the orange color reflects activation of a particular upstream regulator. ERK, extracellular signal–regulated kinase.

Supplemental Figure S6 **A:** Predicted alterations in canonical signaling pathways in human acetaminophen (APAP)-induced acute liver failure (ALF) versus healthy subjects in the second publicly available transcriptomics data set (Gene Expression Omnibus database, *http://www.ncbi.nlm.nih.gov/geo*; accession number GSE120652), identified using Ingenuity Pathway Analysis. **B:** The downstream gene network of MET is predicted to be significantly activated in human ALF. **C** and **D:** Enrichment analysis using DAVID analysis software showing biological processes (Gene Ontology terms) (**C**) and Reactome pathways (**D**) predicted to be altered in human ALF. The number of genes associated with each pathway is indicated on the right side of the corresponding bar. **E:** Upstream regulators predicted to be altered in human ALF were analyzed using Ingenuity Pathway Analysis. **F:** Downstream gene network of hepatocyte growth factor (HGF) is predicted to be significantly activated in human ALF. ERAD, endoplasmic reticulum–associated protein degradation; ERK, extracellular signal–regulated kinase; IRE1, inositol-requiring enzyme 1; PDGF, platelet-derived growth factor.

## References

[1] Stravitz R.T., Fontana R.J., Karvellas C., Durkalski V., McGuire B., Rule J.A., Tujios S., Lee W.M., Acute Liver Failure Study Group (2023). Future directions in acute liver failure. Hepatology.

[2] Ramachandran A., Akakpo J.Y., Curry S.C., Rumack B.H., Jaeschke H. (2024). Clinically relevant therapeutic approaches against acetaminophen hepatotoxicity and acute liver failure. Biochem Pharmacol.

[3] Bhushan B., Apte U. (2019). Liver regeneration after acetaminophen hepatotoxicity: mechanisms and therapeutic opportunities. Am J Pathol.

[4] Jaeschke H., Ramachandran A. (2024). Acetaminophen hepatotoxicity: paradigm for understanding mechanisms of drug-induced liver injury. Annu Rev Pathol.

[5] Bhushan B., Apte U. (2023). Regeneration and recovery after acetaminophen hepatotoxicity. Livers.

[6] Bhushan B., Gunewardena S., Edwards G., Apte U. (2020). Comparison of liver regeneration after partial hepatectomy and acetaminophen-induced acute liver failure: a global picture based on transcriptome analysis. Food Chem Toxicol.

[7] Michalopoulos G.K., Bhushan B. (2021). Liver regeneration: biological and pathological mechanisms and implications. Nat Rev Gastroenterol Hepatol.

[8] Paranjpe S., Bowen W.C., Mars W.M., Orr A., Haynes M.M., DeFrances M.C., Liu S., Tseng G.C., Tsagianni A., Michalopoulos G.K. (2016). Combined systemic elimination of MET and epidermal growth factor receptor signaling completely abolishes liver regeneration and leads to liver decompensation. Hepatology.

[9] Factor V.M., Seo D., Ishikawa T., Kaposi-Novak P., Marquardt J.U., Andersen J.B., Conner E.A., Thorgeirsson S.S. (2010). Loss of c-Met disrupts gene expression program required for G2/M progression during liver regeneration in mice. PLoS One.

[10] Gómez-Quiroz L.E., Factor V.M., Kaposi-Novak P., Coulouarn C., Conner E.A., Thorgeirsson S.S. (2008). Hepatocyte-specific c-Met deletion disrupts redox homeostasis and sensitizes to Fas-mediated apoptosis. J Biol Chem.

[11] Bhushan B., Walesky C., Manley M., Gallagher T., Borude P., Edwards G., Monga S.P.S., Apte U. (2014). Pro-regenerative signaling after acetaminophen-induced acute liver injury in mice identified using a novel incremental dose model. Am J Pathol.

[12] Bhushan B., Chavan H., Borude P., Xie Y., Du K., McGill M.R., Lebofsky M., Jaeschke H., Krishnamurthy P., Apte U. (2017). Dual role of epidermal growth factor receptor in liver injury and regeneration after acetaminophen overdose in mice. Toxicol Sci.

[13] Bhushan B., Stoops J.W., Mars W.M., Orr A., Bowen W.C., Paranjpe S., Michalopoulos G.K. (2019). TCPOBOP-induced hepatomegaly and hepatocyte proliferation are attenuated by combined disruption of MET and EGFR signaling. Hepatology.

[14] Huh C.-G., Factor V.M., Sánchez A., Uchida K., Conner E.A., Thorgeirsson S.S. (2004). Hepatocyte growth factor/c-met signaling pathway is required for efficient liver regeneration and repair. Proc Natl Acad Sci.

[15] Kotulkar M., Paine-Cabrera D., Abernathy S., Robarts D.R., Parkes W.S., Lin-Rahardja K., Numata S., Lebofsky M., Jaeschke H., Apte U. (2023). Role of HNF4alpha-cMyc interaction in liver regeneration and recovery after acetaminophen-induced acute liver injury. Hepatology.

[16] Bhushan B., Molina L., Koral K., Stoops J.W., Mars W.M., Banerjee S., Orr A., Paranjpe S., Monga S.P., Locker J. (2021). Yes-associated protein is crucial for constitutive androstane receptor-driven hepatocyte proliferation but not for induction of drug metabolism genes in mice. Hepatology.

[17] Bano S., Copeland M.A., Stoops J.W., Orr A., Jain S., Paranjpe S., Mooli R.G.R., Ramakrishnan S.K., Locker J., Mars W.M. (2024). Hepatocyte-specific epidermal growth factor receptor deletion promotes fibrosis but has no effect on steatosis in fast-food diet model of metabolic dysfunction-associated steatotic liver disease. Cell Mol Gastroenterol Hepatol.

[18] Park Y., Smith R.D., Combs A.B., Kehrer J.P. (1988). Prevention of acetaminophen-induced hepatotoxicity by dimethyl sulfoxide. Toxicology.

[19] Rutledge C.A., Lagranha C., Chiba T., Redding K., Stolz D.B., Goetzman E., Sims-Lucas S., Kaufman B.A. (2023). Metformin preconditioning protects against myocardial stunning and preserves protein translation in a mouse model of cardiac arrest. J Mol Cell Cardiol Plus.

[20] Tsagianni A., Mars W.M., Bhushan B., Bowen W.C., Orr A., Stoops J., Paranjpe S., Tseng G.C., Liu S., Michalopoulos G.K. (2018). Combined systemic disruption of MET and epidermal growth factor receptor signaling causes liver failure in normal mice. Am J Pathol.

[21] Matchett K.P., Wilson-Kanamori J.R., Portman J.R., Kapourani C.A., Fercoq F., May S. (2024). Multimodal decoding of human liver regeneration. Nature.

[22] Zhao S., Feng Y., Zhang J., Zhang Q., Wang J., Cui S. (2024). Comparative analysis of gene expression between mice and humans in acetaminophen-induced liver injury by integrating bioinformatics analysis. BMC Med Genomics.

[23] Bhushan B., Apte U. (2020). Acetaminophen Test Battery (ATB): a comprehensive method to study acetaminophen-induced acute liver injury. Gene Expr.

[24] Jaeschke H., Adelusi O.B., Akakpo J.Y., Nguyen N.T., Sanchez-Guerrero G., Umbaugh D.S., Ding W.-X., Ramachandran A. (2021). Recommendations for the use of the acetaminophen hepatotoxicity model for mechanistic studies and how to avoid common pitfalls. Acta Pharm Sin B.

[25] Jaeschke H., McGill M.R., Ramachandran A. (2012). Oxidant stress, mitochondria, and cell death mechanisms in drug-induced liver injury: lessons learned from acetaminophen hepatotoxicity. Drug Metab Rev.

[26] Saito C., Lemasters J.J., Jaeschke H. (2010). c-Jun N-terminal kinase modulates oxidant stress and peroxynitrite formation independent of inducible nitric oxide synthase in acetaminophen hepatotoxicity. Toxicol Appl Pharmacol.

[27] Hanawa N., Shinohara M., Saberi B., Gaarde W.A., Han D., Kaplowitz N. (2008). Role of JNK translocation to mitochondria leading to inhibition of mitochondria bioenergetics in acetaminophen-induced liver injury. J Biol Chem.

[28] Park H.-S., Kim M.-S., Huh S.-H., Park J., Chung J., Kang S.S., Choi E.-J. (2002). Akt (protein kinase B) negatively regulates SEK1 by means of protein phosphorylation. J Biol Chem.

[29] Zhao H.-F., Wang J., Tony To S.S. (2015). The phosphatidylinositol 3-kinase/Akt and c-Jun N-terminal kinase signaling in cancer: Alliance or contradiction? (Review). Int J Oncol.

[30] Kim A.H., Yano H., Cho H., Meyer D., Monks B., Margolis B., Birnbaum M.J., Chao M.V. (2002). Akt1 regulates a JNK scaffold during excitotoxic apoptosis. Neuron.

[31] Wang R., Zhang Q.-G., Han D., Xu J., Lü Q., Zhang G.-Y. (2006). Inhibition of MLK3-MKK4/7-JNK1/2 pathway by Akt1 in exogenous estrogen-induced neuroprotection against transient global cerebral ischemia by a non-genomic mechanism in male rats. J Neurochem.

[32] McMillin M., Grant S., Frampton G., Petrescu A.D., Williams E., Jefferson B., DeMorrow S. (2019). The TGF[beta]1 receptor antagonist GW788388 reduces JNK activation and protects against acetaminophen hepatotoxicity in mice. Toxicol Sci.

[33] Bajt M.L., Ramachandran A., Yan H.-M., Lebofsky M., Farhood A., Lemasters J.J., Jaeschke H. (2011). Apoptosis-inducing factor modulates mitochondrial oxidant stress in acetaminophen hepatotoxicity. Toxicol Sci.

[34] Bird T.G., Müller M., Boulter L., Vincent D.F., Ridgway R.A., Lopez-Guadamillas E., Lu W.-Y., Jamieson T., Govaere O., Campbell A.D., Ferreira-Gonzalez S., Cole A.M., Hay T., Simpson K.J., Clark W., Hedley A., Clarke M., Gentaz P., Nixon C., Bryce S., Kiourtis C., Sprangers J., Nibbs R.J.B., Van Rooijen N., Bartholin L., McGreal S.R., Apte U., Barry S.T., Iredale J.P., Clarke A.R., Serrano M., Roskams T.A., Sansom O.J., Forbes S.J. (2018). TGF[beta] inhibition restores a regenerative response in acute liver injury by suppressing paracrine senescence. Sci Transl Med.

[35] Umbaugh D.S., Nguyen N.T., Smith S.H., Ramachandran A., Jaeschke H. (2024). The p21+ perinecrotic hepatocytes produce the chemokine CXCL14 after a severe acetaminophen overdose promoting hepatocyte injury and delaying regeneration. Toxicology.

[36] Umbaugh D.S., Nguyen N.T., Curry S.C., Rule J.A., Lee W.M., Ramachandran A., Jaeschke H., Acute Liver Failure Study Group (2024). The chemokine CXCL14 is a novel early prognostic biomarker for poor outcome in acetaminophen-induced acute liver failure. Hepatology.

[37] Borude P., Bhushan B., Apte U. (2018). DNA damage response regulates initiation of liver regeneration following acetaminophen overdose. Gene Expr.

[38] Uzi D., Barda L., Scaiewicz V., Mills M., Mueller T., Gonzalez-Rodriguez A., Valverde A.M., Iwawaki T., Nahmias Y., Xavier R., Chung R.T., Tirosh B., Shibolet O. (2013). CHOP is a critical regulator of acetaminophen-induced hepatotoxicity. J Hepatol.

[39] Lee A.-H., Scapa E.F., Cohen D.E., Glimcher L.H. (2008). Regulation of hepatic lipogenesis by the transcription factor XBP1. Science.

[40] Buttar H., Nera E., Downie R. (1976). Serum enzyme activities and hepatic triglyceride levels in acute and subacute acetaminophen-treated rats. Toxicology.

[41] Xie Y., McGill M.R., Dorko K., Kumer S.C., Schmitt T.M., Forster J., Jaeschke H. (2014). Mechanisms of acetaminophen-induced cell death in primary human hepatocytes. Toxicol Appl Pharmacol.

[42] Hughes R.D., Zhang L., Tsubouchi H., Daikuhara Y., Williams R. (1994). Plasma hepatocyte growth factor and biliprotein levels and outcome in fulminant hepatic failure. J Hepatol.

[43] Tanabe M., Hosono K., Yamashita A., Ito Y., Majima M., Narumiya S., Kusano C., Amano H. (2024). Deletion of TP signaling in macrophages delays liver repair following APAP-induced liver injury by reducing accumulation of reparative macrophage and production of HGF. Inflamm Regen.

[44] Wang P., Cui Y., Wang J., Liu D., Tian Y., Liu K., Wang X., Liu L., He Y., Pei Y., Li L., Sun L., Zhu Z., Chang D., Jia J., You H. (2022). Mesenchymal stem cells protect against acetaminophen hepatotoxicity by secreting regenerative cytokine hepatocyte growth factor. Stem Cell Res Ther.

[45] Rizvi F., Everton E., Smith A.R., Liu H., Osota E., Beattie M., Tam Y., Pardi N., Weissman D., Gouon-Evans V. (2021). Murine liver repair via transient activation of regenerative pathways in hepatocytes using lipid nanoparticle-complexed nucleoside-modified mRNA. Nat Commun.

