# Supplementary figures and images for: Hepatocyte-Specific MET Deletion Exacerbates Acetaminophen-Induced Hepatotoxicity in Mice
Source: Am J Pathol. 2025 Sep 30;196(2):388–406. doi: 10.1016/j.ajpath.2025.09.010 (PMC12881295; doi:10.1016/j.ajpath.2025.09.010)

A

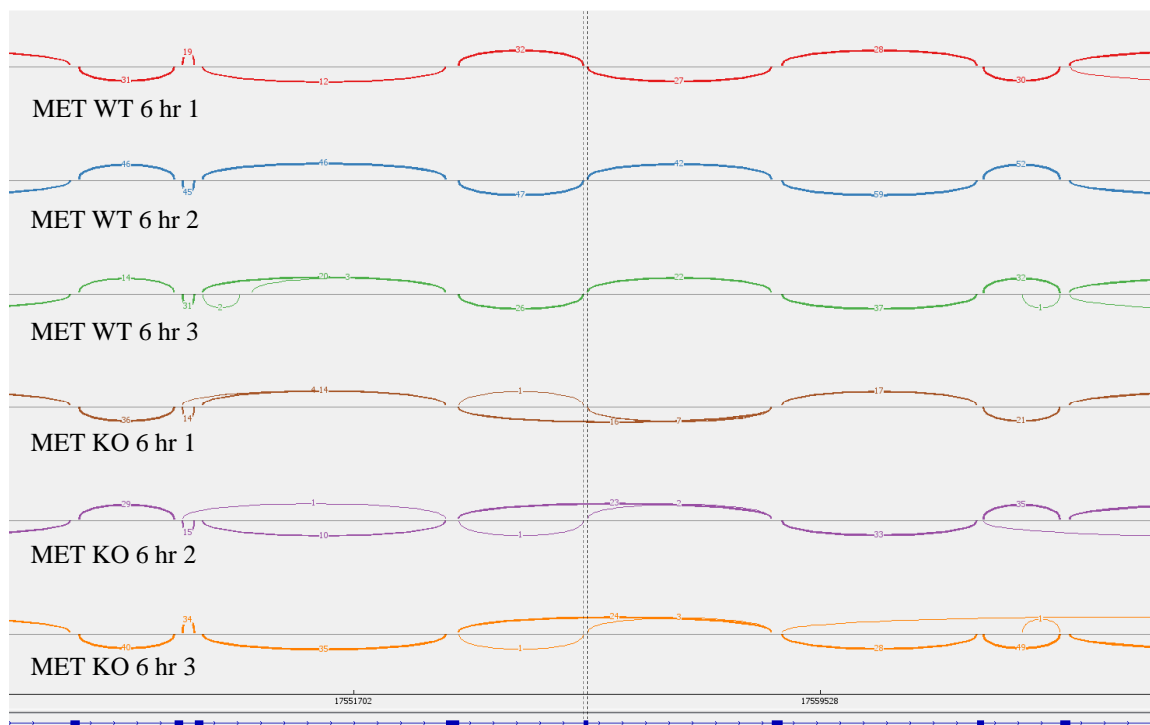

B

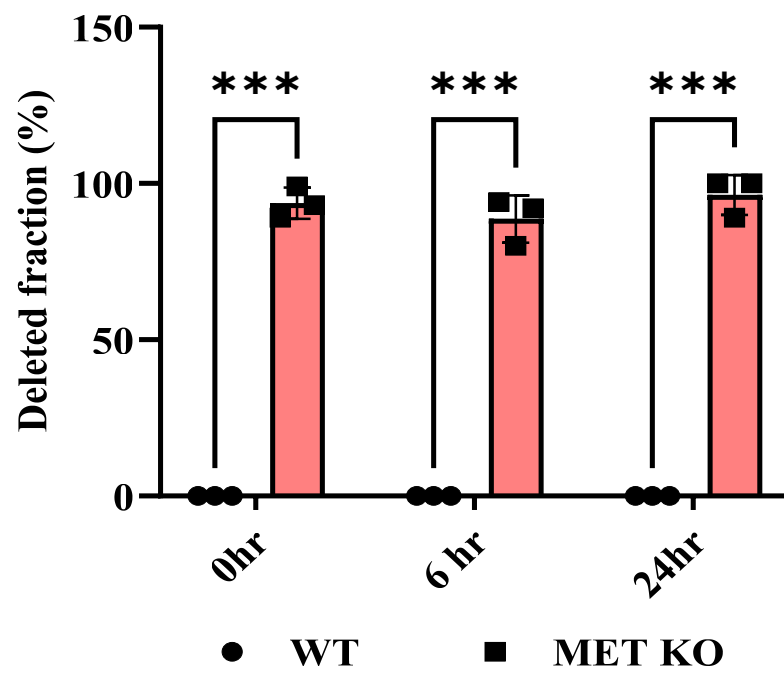

Supplement: Supplemental Figure S1 — Quantification of MET knock-down by AAV8.TBG.PI.Cre.rBG. A: Representative Sashimi plot of RNA-sequencing libraries from MET wild type (WT) and knockout (KO) livers after 6 hours of treatment. The number of intron-spanning reads is marked above the arc that links exon splice sites. The dotted lines mark the position of exon 16, which is deleted in the KO model. The in-frame deletion removes a critical ATP-binding site in the intracellular tyrosine kinase domain, essential for the activation of MET signaling. B: Fraction of spanning reads that join exon 15 to 17, averaged from three livers in each experimental group. ∗∗∗P < 0.001 versus WT mice. [file mmc1.pdf]

A

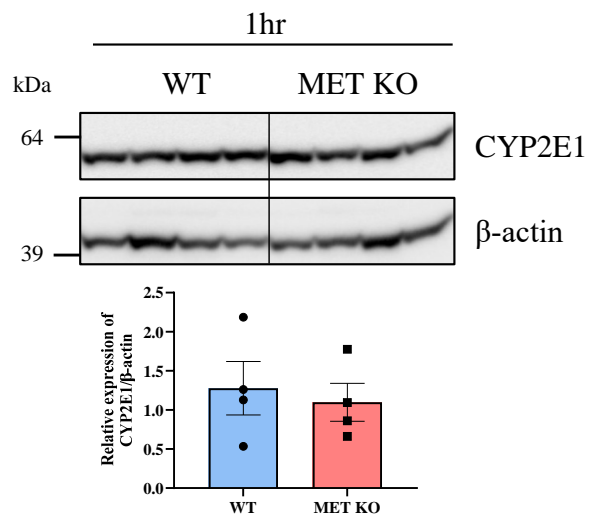

B

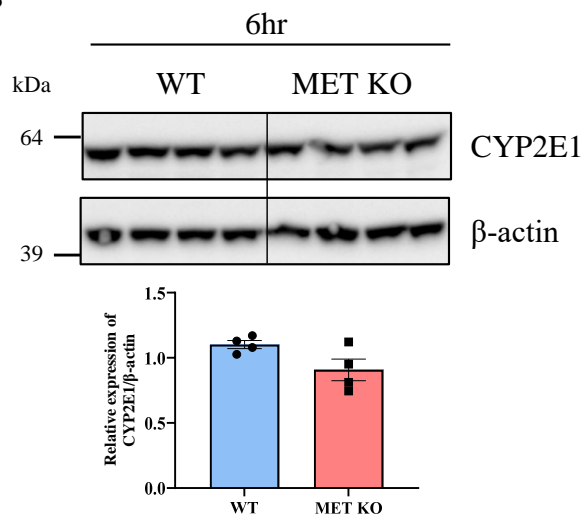

C

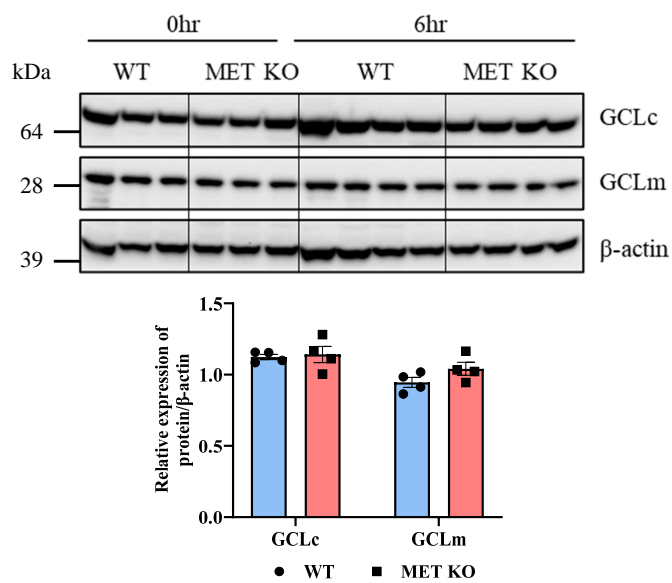

D

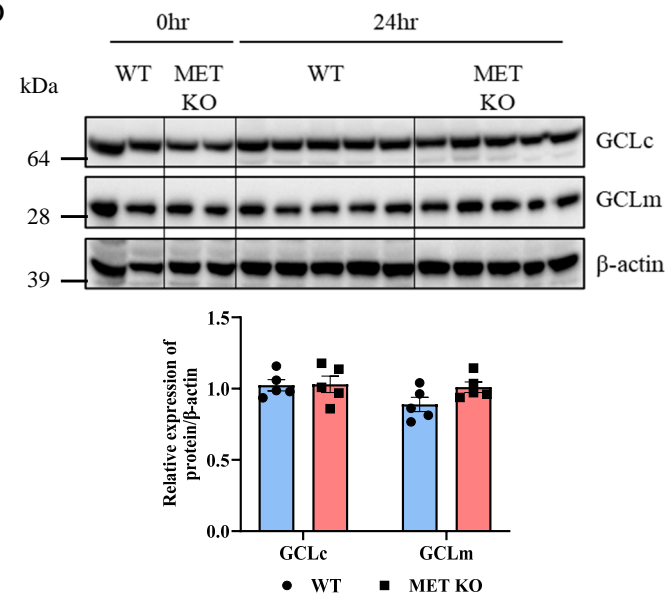

Supplement: Supplemental Figure S2 — A–D: Immunoblot images and densitometric analysis representing the expressions of cytochrome P450 2E1 (CYP2E1) at 1 hour (A) and 6 hours (B) and glutamate-cysteine ligase catalytic subunit (GCLc) and modifier subunit (GCLm) at 6 hours (C) and 24 hours (D) after acetaminophen treatment in wild-type (WT) and MET knockout (KO) mice. [file mmc2.pdf]

**A** *Upstream Regulators Predicted to be Altered in MET-KO vs WT Mice at 6hr*

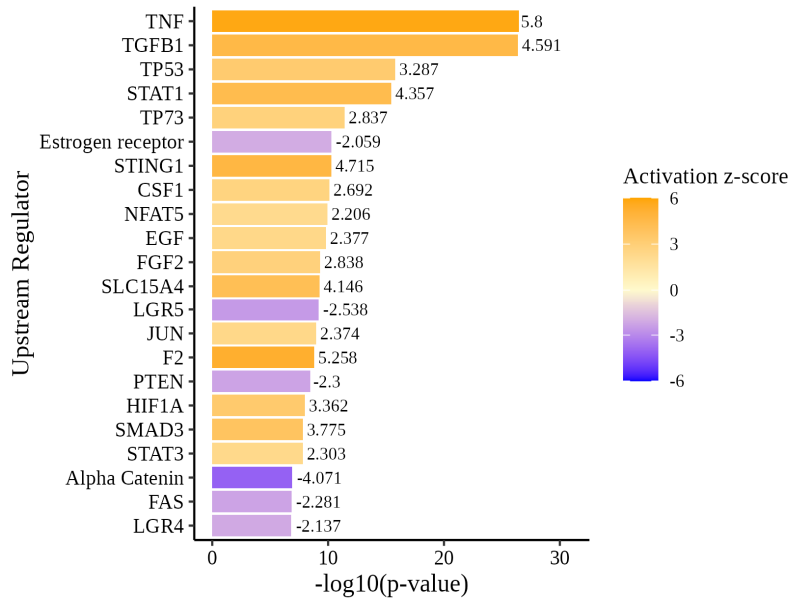

**B**

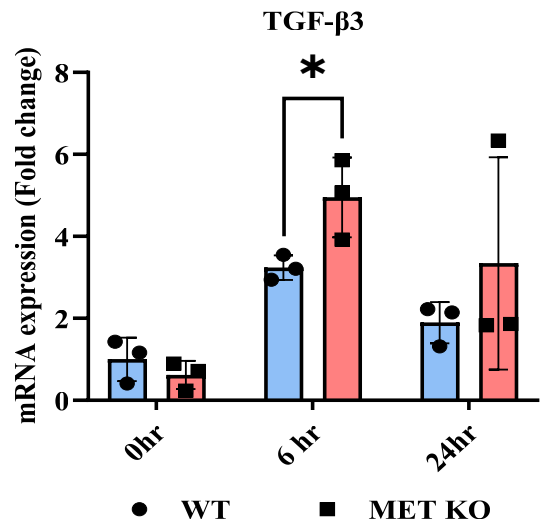

**C**

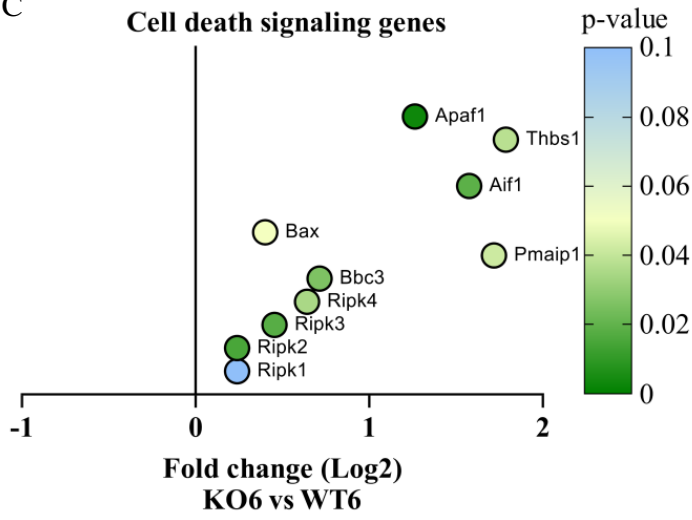

Supplement: Supplemental Figure S3 — A: Upstream regulators predicted to be altered in MET knockout (KO) versus wild-type (WT) mice at 6 hours identified by using Ingenuity Pathway Analysis. Intensity of the blue color reflects extent of inhibition, and the orange color reflects up-regulation of a particular upstream regulator. B: Bar graph representing mRNA expression of transforming growth factor-β3 (TGF-β3). C: Bubble plot representing up-regulation of cell death-associated genes in MET KO versus WT mice at 6 hours (intensity of green color indicates P value significance).∗P < 0.05 versus WT mice. [file mmc3.pdf]
