# Supplementary material for: Hepatocyte-Specific MET Deletion Exacerbates Acetaminophen-Induced Hepatotoxicity in Mice
Source: Am J Pathol. 2025 Sep 30;196(2):388–406. doi: 10.1016/j.ajpath.2025.09.010 (PMC12881295; doi:10.1016/j.ajpath.2025.09.010)
Supplement: Supplemental Figure S4 — A–C: Enrichment analysis using DAVID analysis software showing altered biological processes (Gene Ontology terms) (A), Kyoto Encyclopedia of Genes and Genomes (KEGG) pathways (B), and Reactome pathways (C) in MET knockout (KO) versus wild-type (WT) mice at 24 hours after acetaminophen administration. The number of differentially expressed genes associated with each pathway is indicated on the right side of the corresponding bar. D: Gene Set Enrichment Analysis plot showing depleted “DNA replication” Reactome pathway in MET KO mice versus WT mice at 24 hours. E: Immunoblot images representing the expressions of transforming growth factor-β1 (TGF-β1) and p21 in total liver lysates at 24 hours. F–H: Bar graphs representing mRNA expression of CXCL14 (F), Anxa2 (G), and poly (ADP-ribose) polymerase 1 (PARP1) (H). I: Bubble plot showing the down-regulation of lipogenic genes in MET KO mice at 24 hours (the intensity of the red color indicates P value significance). ∗P < 0.05 and ∗∗∗P < 0.001 versus WT mice. ER, endoplasmic reticulum; ERAD, endoplasmic reticulum–associated protein degradation; NES, normalized enrichment score. [file mmc4.pdf]

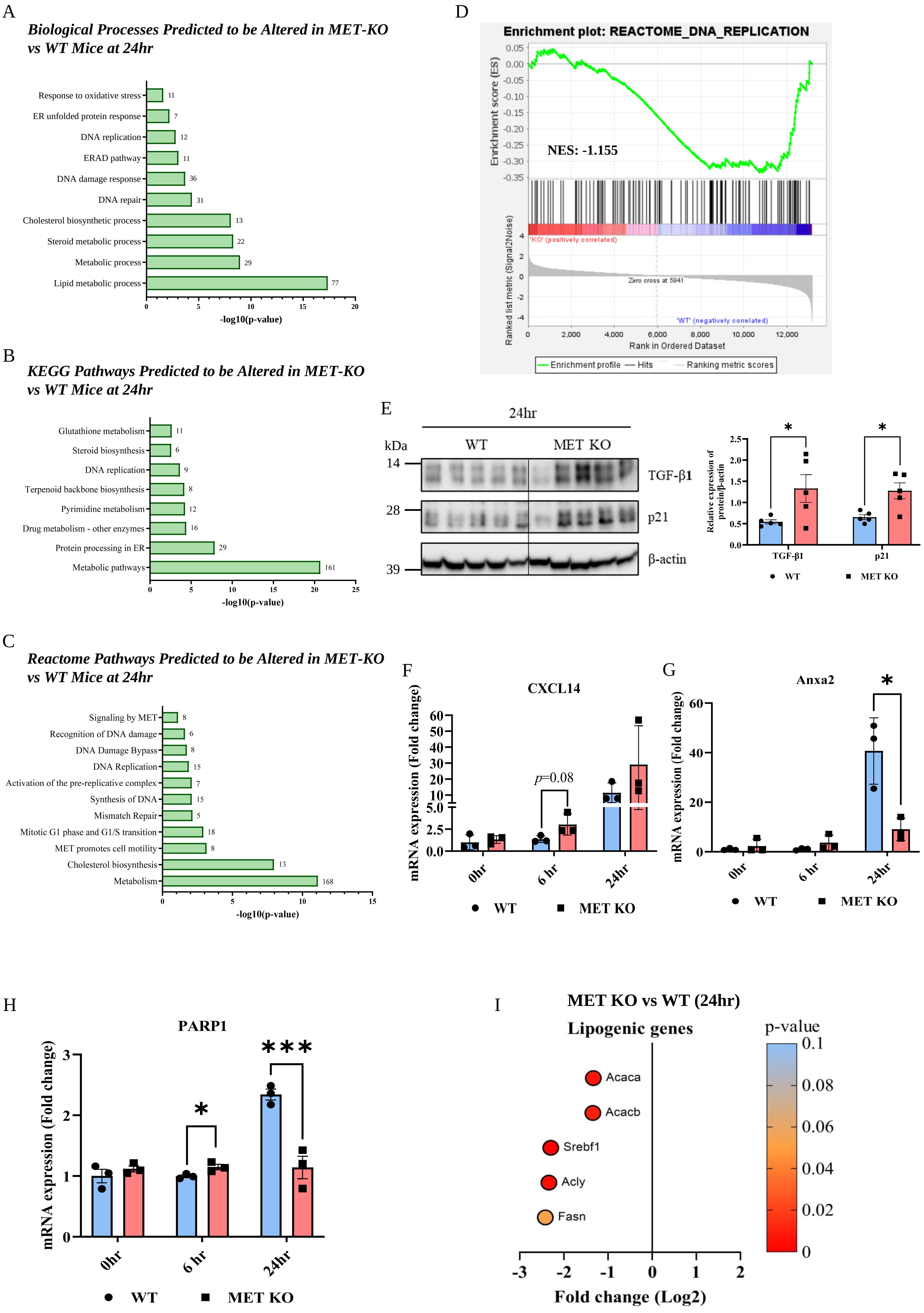
A
Biological Processes Predicted to be Altered in MET-KO vs WT Mice at 24hr
Response to oxidative stress 11
ER unfolded protein response 7
DNA replication 12
ERAD pathway 11
DNA damage response 36
DNA repair 31
Cholesterol biosynthetic process 13
Steroid metabolic process 22
Metabolic process 29
Lipid metabolic process 77
0 5 10 15 20
-log10(p-value)
B
KEGG Pathways Predicted to be Altered in MET-KO vs WT Mice at 24hr
Glutathione metabolism 11
Steroid biosynthesis 6
DNA replication 9
Terpenoid backbone biosynthesis 8
Pyrimidine metabolism 12
Drug metabolism - other enzymes 16
Protein processing in ER 29
Metabolic pathways 161
0 5 10 15 20 25
-log10(p-value)
C
Reactome Pathways Predicted to be Altered in MET-KO vs WT Mice at 24hr
Signaling by MET 8
Recognition of DNA damage 6
DNA Damage Bypass 8
DNA Replication 15
Activation of the pre-replicative complex 7
Synthesis of DNA 15
Mismatch Repair 5
Mitotic G1 phase and G1/S transition 18
MET promotes cell motility 8
Cholesterol biosynthesis 13
Metabolism 168
0 5 10 15
-log10(p-value)
D
Enrichment plot: REACTOME_DNA_REPLICATION
Enrichment score (ES)
NES: -1.155
'KO' (positively correlated)
Zero cross at 5941
'WT' (negatively correlated)
Ranked list metric (Signal2Noise)
Rank in Ordered Dataset
Enrichment profile — Hits — Ranking metric scores
E
24hr
kDa
WT
MET KO
14 TGF-β1
28 p21
39 β-actin
Relative expression of protein/β-actin
TGF-β1
p21
WT
MET KO
F
CXCL14
p=0.08
mRNA expression (Fold change)
0hr
6 hr
24hr
WT
MET KO
G
Anxa2
mRNA expression (Fold change)
0hr
6 hr
24hr
WT
MET KO
H
PARP1
mRNA expression (Fold change)
0hr
6 hr
24hr
WT
MET KO
I
MET KO vs WT (24hr)
Lipogenic genes
Acaca
Acacb
Srebf1
Acly
Fasn
p-value
Fold change (Log2)
