# Supplementary material for: Hepatocyte-Specific MET Deletion Exacerbates Acetaminophen-Induced Hepatotoxicity in Mice
Source: Am J Pathol. 2025 Sep 30;196(2):388–406. doi: 10.1016/j.ajpath.2025.09.010 (PMC12881295; doi:10.1016/j.ajpath.2025.09.010)
Supplement: Supplemental Figure S5 — A: Analysis of publicly available spatial transcriptomics data set (Gene Expression Omnibus database, http://www.ncbi.nlm.nih.gov/geo; accession number GSE223559) showing increased expression of hepatocyte growth factor (HGF) localized around necrotic areas in human acetaminophen (APAP)-induced acute liver failure (ALF) livers. B and C: Enrichment analysis of publicly available single-nuclei RNA sequencing data set (Gene Expression Omnibus database, http://www.ncbi.nlm.nih.gov/geo; accession number GSE223581) using DAVID analysis software showing biological processes (Gene Ontology terms) (B) and Reactome pathways (C) predicted to be altered in human hepatocytes of APAP-ALF versus healthy subjects. The number of genes associated with each pathway is indicated on the right side of the corresponding bar. D: Upstream regulators predicted to be altered in hepatocytes of APAP-ALF patients. Intensity of the blue color reflects extent of inhibition, and the orange color reflects activation of a particular upstream regulator. ERK, extracellular signal–regulated kinase. [file mmc5.pdf]

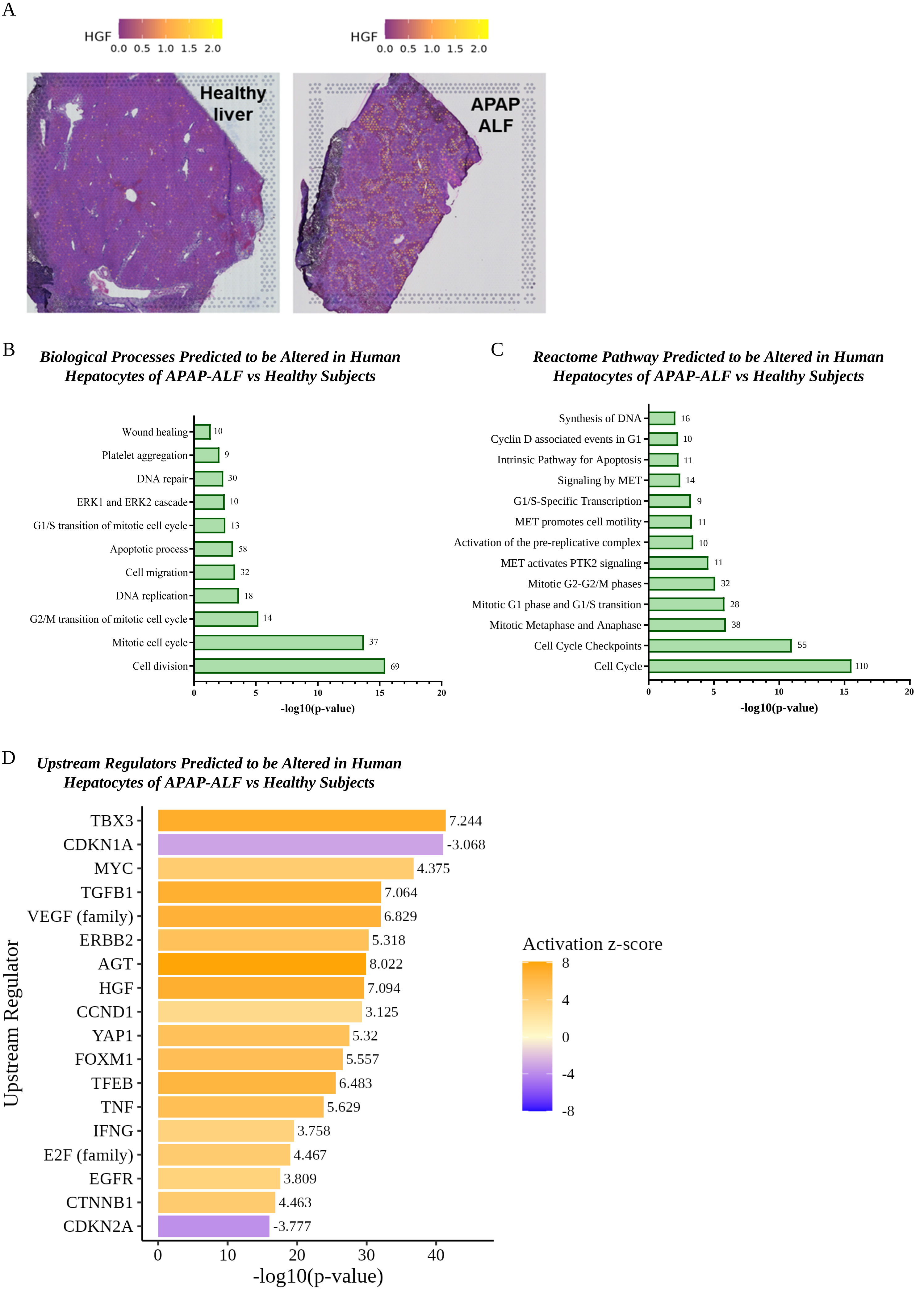
A
HGF
0.0 0.5 1.0 1.5 2.0
HGF
0.0 0.5 1.0 1.5 2.0
Healthy liver
APAP ALF
B
Biological Processes Predicted to be Altered in Human Hepatocytes of APAP-ALF vs Healthy Subjects
Wound healing 10
Platelet aggregation 9
DNA repair 30
ERK1 and ERK2 cascade 10
G1/S transition of mitotic cell cycle 13
Apoptotic process 58
Cell migration 32
DNA replication 18
G2/M transition of mitotic cell cycle 14
Mitotic cell cycle 37
Cell division 69
0 5 10 15 20
-log10(p-value)
C
Reactome Pathway Predicted to be Altered in Human Hepatocytes of APAP-ALF vs Healthy Subjects
Synthesis of DNA 16
Cyclin D associated events in G1 10
Intrinsic Pathway for Apoptosis 11
Signaling by MET 14
G1/S-Specific Transcription 9
MET promotes cell motility 11
Activation of the pre-replicative complex 10
MET activates PTK2 signaling 11
Mitotic G2-G2/M phases 32
Mitotic G1 phase and G1/S transition 28
Mitotic Metaphase and Anaphase 38
Cell Cycle Checkpoints 55
Cell Cycle 110
0 5 10 15 20
-log10(p-value)
D
Upstream Regulators Predicted to be Altered in Human Hepatocytes of APAP-ALF vs Healthy Subjects
Upstream Regulator
TBX3 7.244
CDKN1A -3.068
MYC 4.375
TGFB1 7.064
VEGF (family) 6.829
ERBB2 5.318
AGT 8.022
HGF 7.094
CCND1 3.125
YAP1 5.32
FOXM1 5.557
TFEB 6.483
TNF 5.629
IFNG 3.758
E2F (family) 4.467
EGFR 3.809
CTNNB1 4.463
CDKN2A -3.777
0 10 20 30 40
-log10(p-value)
Activation z-score
8
4
0
-4
-8
