# Supplementary material for: Hepatocyte-Specific MET Deletion Exacerbates Acetaminophen-Induced Hepatotoxicity in Mice
Source: Am J Pathol. 2025 Sep 30;196(2):388–406. doi: 10.1016/j.ajpath.2025.09.010 (PMC12881295; doi:10.1016/j.ajpath.2025.09.010)
Supplement: Supplemental Figure S6 — A: Predicted alterations in canonical signaling pathways in human acetaminophen (APAP)-induced acute liver failure (ALF) versus healthy subjects in the second publicly available transcriptomics data set (Gene Expression Omnibus database, http://www.ncbi.nlm.nih.gov/geo; accession number GSE120652), identified using Ingenuity Pathway Analysis. B: The downstream gene network of MET is predicted to be significantly activated in human ALF. C and D: Enrichment analysis using DAVID analysis software showing biological processes (Gene Ontology terms) (C) and Reactome pathways (D) predicted to be altered in human ALF. The number of genes associated with each pathway is indicated on the right side of the corresponding bar. E: Upstream regulators predicted to be altered in human ALF were analyzed using Ingenuity Pathway Analysis. F: Downstream gene network of hepatocyte growth factor (HGF) is predicted to be significantly activated in human ALF. ERAD, endoplasmic reticulum–associated protein degradation; ERK, extracellular signal–regulated kinase; IRE1, inositol-requiring enzyme 1; PDGF, platelet-derived growth factor. [file mmc6.pdf]

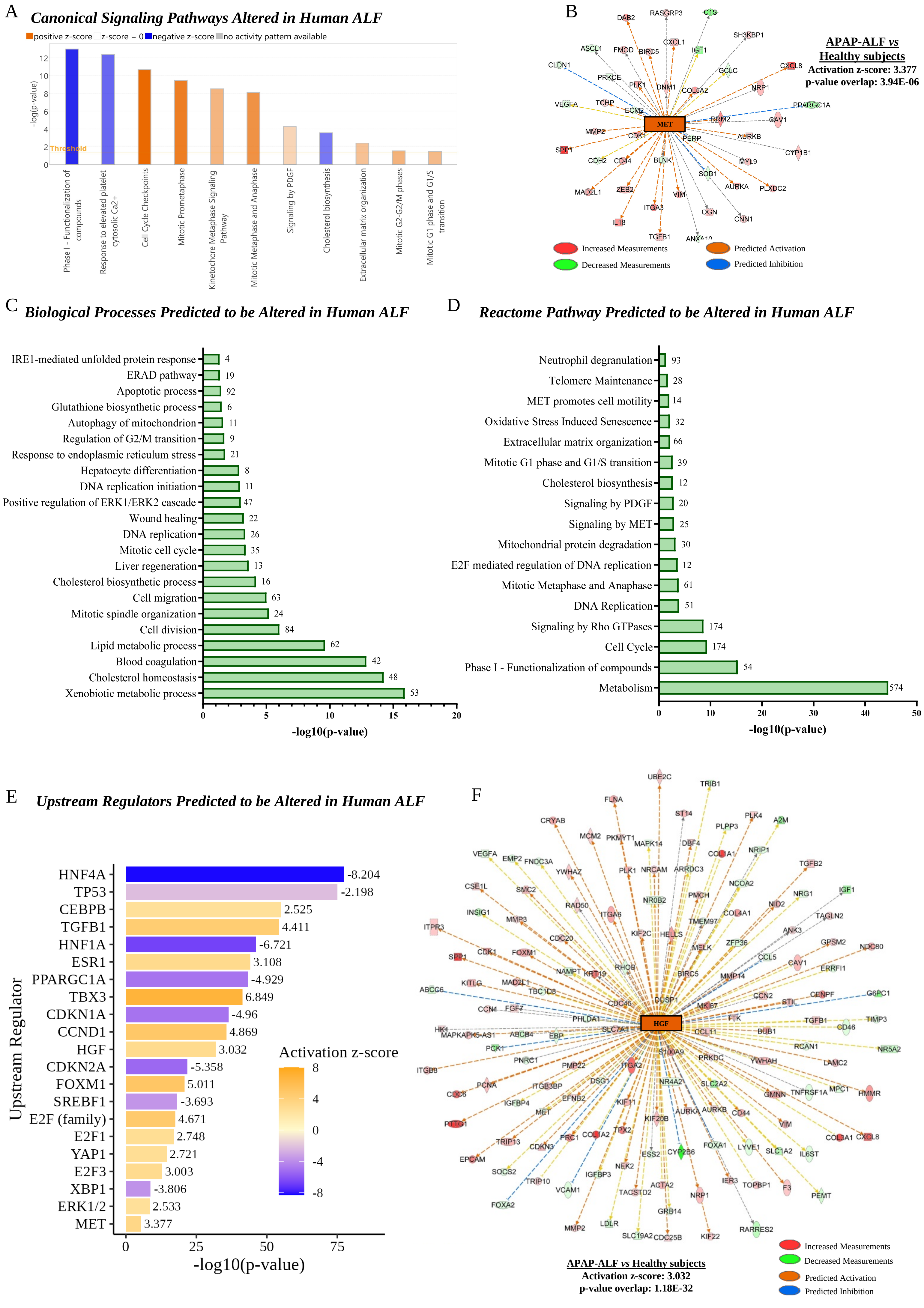
A
Canonical Signaling Pathways Altered in Human ALF
positive z-score
z-score = 0
negative z-score
no activity pattern available
-log(p-value)
Threshold
Phase I - Functionalization of compounds
Response to elevated platelet cytosolic Ca2+
Cell Cycle Checkpoints
Mitotic Prometaphase
Kinetochore Metaphase Signaling Pathway
Mitotic Metaphase and Anaphase
Signaling by PDGF
Cholesterol biosynthesis
Extracellular matrix organization
Mitotic G2-G2/M phases
Mitotic G1 phase and G1/S transition
B
DAB2
RASGRP3
C1S
SH3KBP1
ASCL1
FMOD
BIRC5
CXCL1
IGF1
CLDN1
PRKCE
PLK1
DNM1
COL5A2
GCLC
CXCL8
NRP1
VEGFA
TCHP
ECM2
MET
RRM2
CAV1
PPARGC1A
MMP2
CDK1
PERP
AURKB
SPP1
CYP1B1
CDH2
CD44
BLNK
MYL9
SOD1
MAD2L1
ZEB2
VIM
AURKA
PLXDC2
ITGA3
OGN
CNN1
IL18
TGFB1
APAP-ALF vs Healthy subjects
Activation z-score: 3.377
p-value overlap: 3.94E-06
Increased Measurements
Decreased Measurements
Predicted Activation
Predicted Inhibition
C
Biological Processes Predicted to be Altered in Human ALF
IRE1-mediated unfolded protein response 4
ERAD pathway 19
Apoptotic process 92
Glutathione biosynthetic process 6
Autophagy of mitochondrion 11
Regulation of G2/M transition 9
Response to endoplasmic reticulum stress 21
Hepatocyte differentiation 8
DNA replication initiation 11
Positive regulation of ERK1/ERK2 cascade 47
Wound healing 22
DNA replication 26
Mitotic cell cycle 35
Liver regeneration 13
Cholesterol biosynthetic process 16
Cell migration 63
Mitotic spindle organization 24
Cell division 84
Lipid metabolic process 62
Blood coagulation 42
Cholesterol homeostasis 48
Xenobiotic metabolic process 53
-log10(p-value)
D
Reactome Pathway Predicted to be Altered in Human ALF
Neutrophil degranulation 93
Telomere Maintenance 28
MET promotes cell motility 14
Oxidative Stress Induced Senescence 32
Extracellular matrix organization 66
Mitotic G1 phase and G1/S transition 39
Cholesterol biosynthesis 12
Signaling by PDGF 20
Signaling by MET 25
Mitochondrial protein degradation 30
E2F mediated regulation of DNA replication 12
Mitotic Metaphase and Anaphase 61
DNA Replication 51
Signaling by Rho GTPases 174
Cell Cycle 174
Phase I - Functionalization of compounds 54
Metabolism 574
-log10(p-value)
E
Upstream Regulators Predicted to be Altered in Human ALF
Upstream Regulator
HNF4A -8.204
TP53 -2.198
CEBPB 2.525
TGFB1 4.411
HNF1A -6.721
ESR1 3.108
PPARGC1A -4.929
TBX3 6.849
CDKN1A -4.96
CCND1 4.869
HGF 3.032
CDKN2A -5.358
FOXM1 5.011
SREBF1 -3.693
E2F (family) 4.671
E2F1 2.748
YAP1 2.721
E2F3 3.003
XBP1 -3.806
ERK1/2 2.533
MET 3.377
Activation z-score
-log10(p-value)
F
HGF
APAP-ALF vs Healthy subjects
Activation z-score: 3.032
p-value overlap: 1.18E-32
Increased Measurements
Decreased Measurements
Predicted Activation
Predicted Inhibition
