# Supplementary material for: Hepatocyte-Specific MET Deletion Exacerbates Acetaminophen-Induced Hepatotoxicity in Mice
Source: Am J Pathol. 2025 Sep 30;196(2):388–406. doi: 10.1016/j.ajpath.2025.09.010 (PMC12881295; doi:10.1016/j.ajpath.2025.09.010)
Supplement: Supplemental Table S1 [file mmc7.docx]

**Supplementary Table S1:** **List of downstream genes based on**

**which TGF-β1 was predicted to be activated in MET-KO mice at**

**6hr post-APAP overdose.**

| Genes in dataset downstream of TGF-β1 | Predicted activation status of TGF-β1 (based on measurement direction of downstream gene) | Expr Fold Change  (KO/WT) |
| --- | --- | --- |
| *F13A1* | Activated | 5.553 |
| *PLAT* | Activated | 4.836 |
| *KDELR3* | Activated | 4.49 |
| *FGF21* | Activated | 3.901 |
| *HBEGF* | Activated | 3.807 |
| *THBS1* | Activated | 3.446 |
| *PLAUR* | Activated | 3.272 |
| *FBLN2* | Activated | 3.256 |
| *HTRA1* | Activated | 3.186 |
| *COL16A1* | Activated | 3.028 |
| *SOCS3* | Activated | 2.915 |
| *CCR5* | Activated | 2.679 |
| *SPP1* | Activated | 2.598 |
| *IER3* | Activated | 2.56 |
| *HK2* | Activated | 2.536 |
| *LTBP3* | Activated | 2.53 |
| *PHACTR1* | Activated | 2.511 |
| *CYBA* | Activated | 2.462 |
| *CHST11* | Activated | 2.407 |
| *GDF15* | Activated | 2.354 |
| *RARG* | Activated | 2.325 |
| *TNFAIP3* | Activated | 2.281 |
| *ADAMTS12* | Activated | 2.274 |
| *ELMO1* | Activated | 2.271 |
| *COTL1* | Activated | 2.265 |
| *SERPINH1* | Activated | 2.252 |
| *DOCK2* | Activated | 2.247 |
| *LTBP1* | Activated | 2.218 |
| *HK1* | Activated | 2.21 |
| *PFKP* | Activated | 2.196 |
| *ADAM15* | Activated | 2.171 |
| *MEF2C* | Activated | 2.137 |
| *ISG15* | Activated | 2.137 |
| *ALOX5AP* | Activated | 2.134 |
| *PKM* | Activated | 2.133 |
| *FSCN1* | Activated | 2.126 |
| *COL6A1* | Activated | 2.096 |
| *COL1A2* | Activated | 2.088 |
| *VIM* | Activated | 2.086 |
| *AXL* | Activated | 2.061 |
| *ELK3* | Activated | 2.049 |
| *S1PR3* | Activated | 2.018 |
| *ME2* | Activated | 1.988 |
| *FBN1* | Activated | 1.956 |
| *COL5A1* | Activated | 1.944 |
| *FUT8* | Activated | 1.917 |
| *PDGFRB* | Activated | 1.914 |
| *FILIP1L* | Activated | 1.897 |
| *COL4A1* | Activated | 1.887 |
| *SGK1* | Activated | 1.871 |
| *SOD3* | Activated | 1.859 |
| *SPARC* | Activated | 1.841 |
| *RASGRP3* | Activated | 1.837 |
| *HSPG2* | Activated | 1.796 |
| *GDF10* | Activated | 1.778 |
| *HES1* | Activated | 1.749 |
| *MGMT* | Activated | 1.742 |
| *GLS* | Activated | 1.735 |
| *MCM4* | Activated | 1.728 |
| *MSN* | Activated | 1.723 |
| *LOXL2* | Activated | 1.709 |
| *ZEB2* | Activated | 1.705 |
| *TSPAN7* | Activated | 1.684 |
| *FGFR1* | Activated | 1.683 |
| *RECK* | Activated | 1.668 |
| *DAB2* | Activated | 1.648 |
| *BBC3* | Activated | 1.64 |
| *SELPLG* | Activated | 1.635 |
| *LAMC1* | Activated | 1.628 |
| *CAVIN2* | Activated | 1.625 |
| *ACTN1* | Activated | 1.624 |
| *IFI30* | Activated | 1.599 |
| *ENG* | Activated | 1.585 |
| *TCF7L1* | Activated | 1.582 |
| *APP* | Activated | 1.575 |
| *COL4A2* | Activated | 1.564 |
| *IGF1R* | Activated | 1.522 |
| *PDGFA* | Activated | 1.519 |
| *PTGS1* | Activated | 1.518 |
| *TNFRSF12A* | Activated | 1.517 |
| *ACLY* | Activated | 1.512 |
| *IL4R* | Activated | 1.504 |
| *ESAM* | Activated | 1.502 |
| *NOTUM* | Activated | -1.581 |
| *GNA14* | Activated | -1.997 |
| *APOC2* | Activated | -2.682 |
