# Supplementary material for: Hepatocyte-Specific MET Deletion Exacerbates Acetaminophen-Induced Hepatotoxicity in Mice
Source: Am J Pathol. 2025 Sep 30;196(2):388–406. doi: 10.1016/j.ajpath.2025.09.010 (PMC12881295; doi:10.1016/j.ajpath.2025.09.010)
Supplement: Supplemental Table S2 [file mmc8.docx]

**Supplementary Table S2:** **List of downstream genes based on**

**which XBP1 was predicted to be inhibited in MET-KO mice at**

**24hr post-APAP overdose.**

| Genes in dataset downstream of XBP1 | Prediction activation status of XBP1 (based on measurement direction of downstream gene) | Expr Fold Change  (KO/WT) |
| --- | --- | --- |
| *TXNDC11* | Inhibited | -1.54 |
| *COG6* | Inhibited | -1.549 |
| *SPCS3* | Inhibited | -1.575 |
| *LMAN1* | Inhibited | -1.666 |
| *SSR1* | Inhibited | -1.686 |
| *PDIA5* | Inhibited | -1.704 |
| *COPG1* | Inhibited | -1.737 |
| *GALK2* | Inhibited | -1.737 |
| *SEC61A1* | Inhibited | -1.738 |
| *SYVN1* | Inhibited | -1.745 |
| *HSPA13* | Inhibited | -1.748 |
| *CDK5RAP3* | Inhibited | -1.799 |
| *RPN1* | Inhibited | -1.804 |
| *UBA5* | Inhibited | -1.844 |
| *RPN2* | Inhibited | -1.89 |
| *MCFD2* | Inhibited | -1.9 |
| *PIGA* | Inhibited | -1.908 |
| *SPCS2* | Inhibited | -1.913 |
| *LMAN2* | Inhibited | -1.923 |
| *CALR* | Inhibited | -1.926 |
| *SEC23B* | Inhibited | -1.987 |
| *DNAJB11* | Inhibited | -1.995 |
| *COPZ1* | Inhibited | -2.025 |
| *COPE* | Inhibited | -2.074 |
| *PDIA4* | Inhibited | -2.123 |
| *RCN3* | Inhibited | -2.149 |
| *PPIB* | Inhibited | -2.208 |
| *SELENOM* | Inhibited | -2.209 |
| *DDOST* | Inhibited | -2.301 |
| *ESR1* | Inhibited | -2.332 |
| *EXTL2* | Inhibited | -2.432 |
| *SEC61B* | Inhibited | -2.466 |
| *HYOU1* | Inhibited | -2.514 |
| *TXNDC5* | Inhibited | -2.646 |
| *OSTC* | Inhibited | -2.682 |
| *EDEM1* | Inhibited | -2.732 |
| *SEC61G* | Inhibited | -2.74 |
| *GMPPB* | Inhibited | -2.955 |
| *FKBP11* | Inhibited | -3.163 |
| *GGCX* | Inhibited | -3.243 |
| *PDIA6* | Inhibited | -3.679 |
| *BHLHA15* | Inhibited | -4.306 |
| *SREBF1* | Inhibited | -4.919 |
| *2L1* | Inhibited | -4.98 |
